# An integrated intervention for chronic care management in rural Nepal: protocol of a type 2 hybrid effectiveness-implementation study

**DOI:** 10.1186/s13063-020-4063-3

**Published:** 2020-01-29

**Authors:** Dan Schwarz, Santosh Dhungana, Anirudh Kumar, Bibhav Acharya, Pawan Agrawal, Anu Aryal, Aaron Baum, Nandini Choudhury, David Citrin, Binod Dangal, Meghnath Dhimal, Bikash Gauchan, Tula Gupta, Scott Halliday, Biraj Karmacharya, Sandeep Kishore, Bhagawan Koirala, Uday Kshatriya, Erica Levine, Sheela Maru, Pragya Rimal, Sabitri Sapkota, Ryan Schwarz, Archana Shrestha, Aradhana Thapa, Duncan Maru

**Affiliations:** 1 Nyaya Health Nepal, Kathmandu, Nepal; 2 Division of Global Health Equity, Department of Medicine, Brigham and Women’s Hospital, 75 Francis Street, Boston, MA USA; 3 Department of Medicine, Harvard Medical School, Boston, MA USA; 4 Department of Medicine, Beth Israel Deaconess Medical Center, Boston, MA USA; 5 Ariadne Labs, Harvard T.H. Chan School of Public Health and Brigham and Women’s Hospital, Boston, MA USA; 6 Department of Internal Medicine, Hurley Medical Center, Flint, MI USA; 7 Department of Medicine, NYU Langone Health, New York, NY USA; 8 Department of Psychiatry, University of California San Francisco, San Francisco, CA USA; 9 School of Medical Sciences, Kathmandu University, Dhulikhel, Nepal; 10 Arnhold Institute for Global Health, Icahn School of Medicine at Mount Sinai, New York, NY USA; 11 Department of Global Health, University of Washington, Seattle, WA USA; 12 Department of Anthropology, University of Washington, Seattle, WA USA; 13 Henry M. Jackson School of International Studies, University of Washington, Seattle, WA USA; 14 Nepal Health Research Council, Ministry of Health and Population, Kathmandu, Nepal; 15 Health Equity Action Leadership Initiative, University of California, San Francisco, San Francisco, CA USA; 16 Nepal Technology Innovation Center, Kathmandu University, Dhulikhel, Nepal; 17 Sun Yat-sen Global Health Institute, Sun Yat-sen University, Guangzhou, China; 18 Department of Health Systems Design and Global Health, Icahn School of Medicine at Mount Sinai, New York, NY USA; 19 Young Professionals Chronic Disease Network, New York, NY USA; 20 Institute of Medicine, Tribhuvan University Teaching Hospital, Kathmandu, Nepal; 21 Department of Obstetrics, Gynecology, and Reproductive Sciences, Icahn School of Medicine at Mount Sinai, New York, NY USA; 22 Division of General Internal Medicine, Department of Medicine, Massachusetts General Hospital, Boston, MA USA; 23 Yale School of Public Health, Center for Methods in Implementation and Prevention Science, New Haven, CT USA; 24 Department of Chronic Disease Epidemiology, Yale School of Public Health, New Haven, CT USA; 25 Department of Internal Medicine, Icahn School of Medicine at Mount Sinai, New York, NY USA; 26 Department of Pediatrics, Icahn School of Medicine at Mount Sinai, New York, NY USA

**Keywords:** Noncommunicable diseases, Chronic illness, Community health workers, Decision support systems, Motivational interviewing, Rural health, Nepal

## Abstract

**Background:**

In Nepal, the burden of noncommunicable, chronic diseases is rapidly rising, and disproportionately affecting low and middle-income countries. Integrated interventions are essential in strengthening primary care systems and addressing the burden of multiple comorbidities. A growing body of literature supports the involvement of frontline providers, namely mid-level practitioners and community health workers, in chronic care management. Important operational questions remain, however, around the digital, training, and supervisory structures to support the implementation of effective, affordable, and equitable chronic care management programs.

**Methods:**

A 12-month, population-level, type 2 hybrid effectiveness-implementation study will be conducted in rural Nepal to evaluate an integrated noncommunicable disease care management intervention within Nepal’s new municipal governance structure. The intervention will leverage the government’s planned roll-out of the World Health Organization’s Package of Essential Noncommunicable Disease Interventions (WHO-PEN) program in four municipalities in Nepal, with a study population of 80,000. The intervention will leverage both the WHO-PEN and its cardiovascular disease-specific technical guidelines (HEARTS), and will include three evidence-based components: noncommunicable disease care provision using mid-level practitioners and community health workers; digital clinical decision support tools to ensure delivery of evidence-based care; and training and digitally supported supervision of mid-level practitioners to provide motivational interviewing for modifiable risk factor optimization, with a focus on medication adherence, and tobacco and alcohol use. The study will evaluate effectiveness using a pre–post design with stepped implementation. The primary outcomes will be disease-specific, “at-goal” metrics of chronic care management; secondary outcomes will include alcohol and tobacco consumption levels.

**Discussion:**

This is the first population-level, hybrid effectiveness-implementation study of an integrated chronic care management intervention in Nepal. As low and middle-income countries plan for the Sustainable Development Goals and universal health coverage, the results of this pragmatic study will offer insights into policy and programmatic design for noncommunicable disease care management in the future.

**Trial registration:**

ClinicalTrials.gov, NCT04087369. Registered on 12 September 2019.

## Background

The burden of noncommunicable diseases (NCDs) is rising globally [1, 2], and four major NCD classes—cardiovascular disease, chronic respiratory disease, diabetes, and cancer—contribute to more deaths globally than all other diseases combined, with enormous health and economic implications currently and in the future [3, 4]. This is especially true in low and middle-income countries (LMICs), where governments are struggling to plan for the increasing health and financial implications of these pandemics [5]. As governments strive to achieve the Sustainable Development Goals [6] and work toward universal health coverage [7], the rising NCD pandemic poses significant barriers to these aspirations.

In spite of this growing need for long-term chronic disease care, LMIC healthcare systems are not well equipped to address this disease burden [8]. Historically focused on delivering acute, episodic care, these healthcare systems lack the capacity to provide ongoing longitudinal care for patients with diseases best managed across a lifetime. This is especially true for patients with multiple chronic conditions, who often struggle to receive care for comorbid diseases without numerous, fragmented encounters [9]. Health workforce shortages and an emphasis on physician-provided care have contributed to significant access, coverage, and quality deficits [10, 11].

There is a growing body of evidence that chronic diseases in LMICs can be well managed, especially at the primary care level, by mid-level practitioners (MLPs) [10, 12–21]. MLPs offer a feasible, affordable, and high-quality alternative to traditionally physician-delivered care. This can help to circumvent access barriers, thereby leading to fewer late-stage presentations and complications of otherwise manageable chronic conditions [20]. Evidence suggests that the quality of MLP-delivered care improved when they are well-managed, assigned clear scopes of work for which they are appropriately trained, and receive supportive supervision [22–24] from senior clinicians with continual, improvement-focused feedback [20].

Simultaneously, the role of community health workers (CHW) is increasingly recognized as an important augmentation to facility-based care delivery strategies, offering critical linkages, referrals, and coordination of care within communities [25–27]. The potential value of CHW-augmented chronic care management has only recently become of major interest [12–18, 27–29]. Global experience suggests that the greatest benefits come from CHWs when they are accredited, well-managed, salaried, continually trained and supervised, integrated into strong primary care systems, and part of continual data feedback loops with facility-based providers [25].

Taken together, the need and opportunity for LMIC healthcare systems—already struggling with workforce shortages—to build strong chronic care programs managed by MLPs and augmented with CHWs are compelling. However, there is a paucity of large-scale implementation data available to evaluate such models of care delivery, making it difficult for LMIC policy-makers to decide whether to adopt these strategies.

In response to the growing need for evidence-based NCD service delivery, the World Health Organization has compiled a set of protocols within the Package of Essential Noncommunicable Disease Interventions (WHO-PEN) for primary health care in low-resource settings [30, 31]. These protocols constitute simple decision-making algorithms for the screening, diagnosis, and management of major NCDs, helping to ensure quality care provision in primary care settings, especially in areas already struggling with workforce shortages.

The WHO, together with the World Heart Federation, the World Stroke Organization, the United States Centers for Disease Control, the International Society of Hypertension, and the World Hypertension League, has released complementary guidelines to the WHO-PEN, called HEARTS [32]. (The acronym stands for: Healthy-lifestyle counselling. Evidence-based treatment protocols. Access to essential medicines and technology. Risk-based charts. Team-based care. Systems for monitoring.) In addition to bringing specific guidelines for cardiovascular disease management into the technical base of WHO-PEN, these guidelines provide expanded details around health information systems, workforce development and training, and service delivery. As such, they augment the systems focus of WHO-PEN in incorporating NCD care into broader healthcare systems strengthening.

Recent evidence has shown the feasibility of adopting WHO-PEN at the primary care level in LMICs, including implementation with MLPs [33–36]. These data suggest that it is feasible to deploy WHO-PEN at the population level in primary care settings. While WHO-PEN and HEARTS do not focus explicitly on the engagement of CHWs in these care delivery chains, there is a strong potential for augmenting MLP-based WHO-PEN and HEARTS care at facilities with CHW-based strategies in the community.

Clinical decision support (CDS) tools facilitate the use of algorithmic care protocols such as WHO-PEN by health workers at the point of care. HEARTS provides specific CDS algorithms for cardiovascular conditions. These algorithmic CDS tools are used globally, across high-income and LMIC settings, in a range of conditions, including heart disease and diabetes [37–42]. CDS tools are essential both for managing individual conditions as well as for structuring the comprehensive care of patients with multiple comorbidities. In addition to CDS tools integrated into electronic health records (EHRs), there is a growing body of evidence to support mobile-phone-based CDS tools, often targeted at MLPs [43] and CHWs [44]. These CDS tools are especially well equipped for clinical situations in which clear algorithms exist, such as the WHO-PEN and HEARTS protocols.

Historically, in many LMICs, there has been scant emphasis placed on risk factor reduction through lifestyle modification for NCD prevention or to reduce risk factors for the progression of these diseases when already diagnosed. While many of the contributing etiologies for NCD epidemics are far upstream of individuals’ lives, and out of their control [45], there are certain risk factors that are modifiable by patients, families, and health workers. Among others, it is widely documented that alcohol and tobacco consumption directly contribute to the development and progression of cardiovascular disease, diabetes, and chronic obstructive pulmonary disease (COPD) [46]. These risk factors have been marked as priority targets for NCD control moving forward, including in both the WHO-PEN and HEARTS protocols [8, 47].

WHO-PEN Protocol #2 (republished in HEARTS), “*Health Education and Counseling on Healthy Behaviors*,” describes risk factor modification [31]. However, while emphasizing health education, there is often very little guidance provided to health workers for meaningful engagement with patients and communities to accomplish these difficult lifestyle modification goals. There are many limitations to purely knowledge-focused approach health education, versus one that emphasizes self-efficacy and patients’ own values [48].

Motivational interviewing (MI), originally validated in substance-abuse interventions, approaches behavior change through a style characterized by empathy and collaboration aimed toward the patient’s readiness for change [49]. Trial data demonstrate the efficacy of MI for behavior change such as smoking and alcohol cessation [50–55]. These data raise the possibility of MI program development, led by MLPs within LMICs, as a viable strategy for improving risk factor modification interventions, and, specifically, as an augmentation to WHO-PEN, to strengthen its impact. Since many communities struggle with a lack of healthy nutritional options, dietary improvements are often difficult to realize. Reducing alcohol and tobacco consumption, however, are realistic options for risk factor modification interventions in even the poorest communities worldwide. Recent WHO-PEN guidance on “brief interventions” for alcohol and tobacco, in concert with MI-related interventions, offer practical options for addressing these challenges in the primary care setting [48].

In Nepal, the burden of NCDs is rapidly growing [56–58], within the context of an overburdened healthcare system [12, 58, 59]. Nepal’s government is committed to achieving the Sustainable Development Goals and universal healthcare, and has a specific focus on expanding health services for NCDs. In addition to recently enshrining the right to healthcare into its Constitution [60], Nepal’s government has committed to addressing the NCD epidemic by launching a Multisectoral Action Plan in 2014 [61] and establishing the Nepal Noncommunicable Disease & Injury Poverty Commission in 2016 [58]. Furthermore, the Ministry of Health and Population has committed to a step-wise national implementation of PEN [58, 59, 62]. Nonetheless, the way forward will be challenging, and innovative strategies are clearly needed to accomplish these ambitious goals [58].

Nepal has an extensive history of both MLP and CHW interventions [63–65]. Recent evidence from within the country has demonstrated the potential for CHWs to be involved in hypertension management [66]. Many communities rely on MLPs for primary care [43, 67], and there has been some experience with MLPs using CDS tools for algorithmic care provision [43]. However, to date there are no large-scale implementation data for integrated, MLP-based and CHW-based NCD care management at the population level. Similarly, while there has been some anecdotal description of MI being utilized in urban areas for select populations, there is no population-level data surrounding the use of MI for NCD risk modification. As the country endeavors to develop cross-sectoral strategies to address the growing NCD epidemic, these staffing models, coupled with CDS tools and MI-based adherence and risk modification, are important policy and programmatic considerations.

We will conduct a type 2 hybrid effectiveness-implementation trial (where effectiveness and implementation are simultaneously tested with equal priority simultaneously) [68, 69] to evaluate an integrated NCD care management intervention. The intervention will leverage the Nepali government’s planned roll-out of WHO-PEN in two rural districts. In addition to the government’s roll-out, the intervention will include three evidence-based components: NCD care provision by MLPs and CHWs that is integrated between facilities and communities; CDS tools for MLPs and CHWs to optimize adherence to best practices; and training and supervision of MLPs in using MI to facilitate tobacco and alcohol cessation.

## Methods

### Study aims

As already stated, we will conduct a type 2 hybrid effectiveness-implementation trial to evaluate an integrated NCD care management intervention in rural Nepal. The intervention is described in depth in Additional file 1.

### Study implementers

Healthcare workers and research staff from the nonprofit organization Nyaya Health Nepal, their collaborators in the Ministry of Health and Population and Nepal Health Research Council, and collaborating researchers form the study team will lead the study. Nyaya Health Nepal has been working in a public–private partnership with the Ministry of Health and Population for over 10 years in rural Nepal to deliver community-based and facility-based health services, and this study will leverage this pre-existing partnership and care delivery network. Nyaya Health Nepal operates with a US-based nonprofit organization, Possible, to advance national and global healthcare systems policy and practice priorities.

### Study setting

The study will take place in the Achham and Dolakha districts of Nepal across four municipalities. Following recent healthcare decentralization, Nepal’s 750 municipalities manage primary healthcare delivery. The intervention will be implemented in a step-wise fashion in coordination with municipal-level government authorities and study staff.

Achham is a remote, impoverished district of 260,000 people, with large migrant populations and a history of social disruption during the Nepali civil conflict [70–74]. Achham has one of the highest district-level under-5 mortality rates [75] and one of the lowest human development indices in the country [76]. The study implementers have been delivering some NCD-related care at the district-level Bayalpata Hospital and to communities within the hospital’s catchment population since 2008. Bayalpata Hospital serves approximately 90,000 outpatient and 3000 inpatient visits per year. CHW services include proactive case detection, care coordination, and counseling. The study will include a catchment population of approximately 50,000 in Achham across two municipalities.

The second district is Dolakha, one of the hardest hit districts in the 2015 earthquakes [77]. Nyaya Health Nepal’s work in Dolakha is based at Charikot Primary Health Care Center, which serves approximately 60,000 outpatients per year, with similar CHW services to those in Achham’s. The study will include a population of approximately 30,000 in Dolakha across two municipalities. Thus, the total expected study population will be 80,000.

Within the context of the public–private partnership between the government and Nyaya Health Nepal, no user fees are charged for any facility-based or community-based services, in either Achham or Dolakha, thereby mitigating financial access barriers to care delivery and study participation.

Within the study setting, MLPs for the NCD intervention are locally defined as the Nepali cadre of health assistants, who have 3 years of postsecondary medical training. The CHWs in this intervention have secondary-school-level education and are fully employed, with ongoing supervision from community health nurses (CHNs). They receive initial training of approximately 1 month when they are hired, and ongoing weekly trainings to continually improve their skillsets. The CHWs are employed by the public–private partnership between Nyaya Health Nepal and the Ministry of Health and Population. They are distinct from the robust Female Community Health Volunteer network that exists throughout Nepal [63, 78], who have historically focused on vaccination, public health messaging, and other community preventive interventions rather than on household care delivery and follow-up. These staffing, supervision, and training structures are described in greater detail in Additional files 1 and 2.

### Study populations

For primary quantitative outcomes, the study population will include adult patients (≥ 18 years of age) who qualify for a diagnosis of hypertension, type II diabetes, and/or COPD, according to WHO-PEN guidelines, and are engaged in longitudinal care by Nyaya Health Nepal’s team in Achham and Dolakha. The study will limit enrollment to the catchment areas served by both the facility-level and CHW-level services deployed by Nyaya Health Nepal. Study participants will be initially enrolled during facility-based visits at Bayalpata Hospital and Charikot Primary Health Care Center prior to the completion of intervention roll-out, and are considered engaged in longitudinal care if they have at least one follow-up hospital visit after 12 months of their initial visit. Digital health records that link between the facility-based EHR and the CHWs’ mobile-phone applications will be utilized to share patient data across settings, when available. CHWs can identify potential patients in the community and refer them to the facility for diagnosis confirmation, following which they could be included in the study. Patients’ receipt of care will not be contingent upon their enrollment in the study; all patients will continue to receive care per routine service delivery. This represents an exhaustive convenience sampling method as all eligible patients identified at Bayalpata Hospital and Charikot Primary Health Care Center may be enrolled in the study. Exclusion criteria are individuals planning to migrate from the study area prior to 12 months of exposure to the intervention, or individuals explicitly requesting exclusion from the study or declining to consent (see Additional file 3) for the study.

For implementation components, staff members, patients, community leaders, and government officials will be approached for key-informant interviews (KIIs) and focus group discussions (FGDs), as described in the following.

### Study design

This is a prospective, mixed-methods type 2 hybrid effectiveness-implementation study to evaluate an integrated NCD care management intervention. We plan to apply both qualitative and quantitative methods in a complementary manner [79], in order to meaningfully assess both patient-level and population-level outcomes and the effectiveness of the implementation strategy. We will study the intervention’s impact on patients’ disease management outcomes after 12 months of being enrolled in NCD care using a pre–post design across both sites:

1. We will study the implementation of the intervention utilizing both quantitative and qualitative methods applying the RE-AIM (Reach, Effectiveness, Adoption, Implementation, Maintenance) framework [80].

**Fig. 1:**
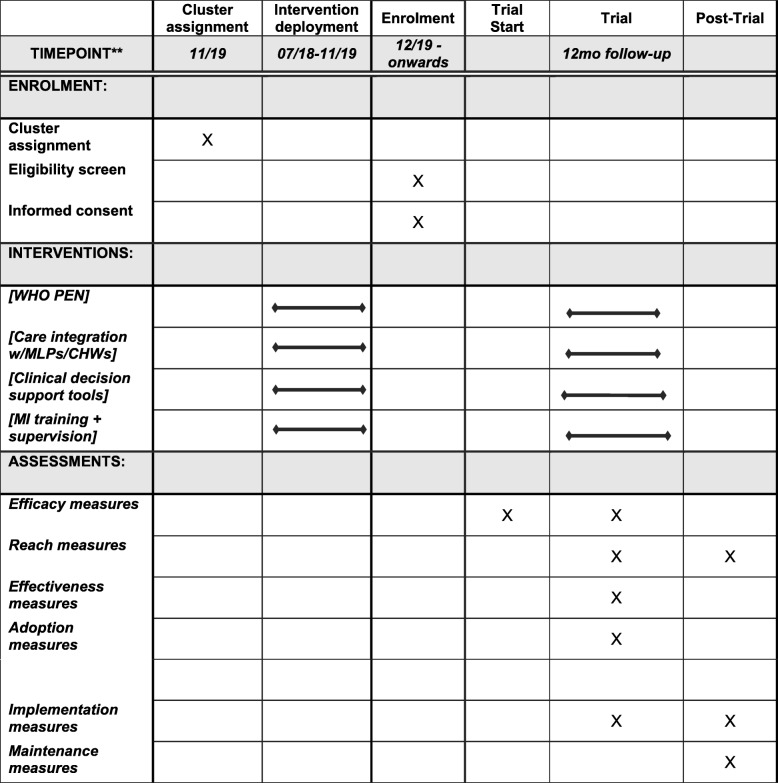
Standard Protocol Items: Recommendations for Interventional Trials (SPIRIT) figure. [81] Recommended content can be displayed using various schematic formats. See SPIRIT 2013 Explanation and Elaboration for examples from protocols. **List specific timepoints in this row

Data collection is developed and integrated within the routine course of delivering care, which is an ethical, acceptable, and affordable approach in this setting. See Fig. 1 for a trial flowchart and Additional file 4 for a SPIRIT research reporting checklist. It is not feasible nor ethically acceptable to obtain data on a comparison (control) group in this population. Given the lack of national or local NCD systems, no data are available from other sources prior to the start of the study.

### Study outcomes

The study has two specific aims: effectiveness (Specific Aim 1) and implementation (Specific Aim 2), as detailed in Table 1. For Specific Aim 1, the primary outcome will be the proportion of patients who meet disease-specific, evidence-based control measures at the completion of their initial 12 months engaged in treatment. These “at-goal” metrics aim to serve as simplified measures to assess disease control status, recognizing the limitations associated with multiple disease-specific metrics in settings like rural Nepal, especially for patients with multiple comorbid conditions. These are presented in Table 2.

**Table 1:** Metrics for Specific Aim 1 (efficacy) and Specific Aim 2 (implementation)

Aim	Outcome/RE-AIM element	Indicator	Definition
Specific Aim 1: efficacy	Primary outcome: control of NCD conditions	Condition-specific “at-goal” metrics	% of enrolled NCD patients achieving “at-goal” status (Table 2), at completion of the study period
	Secondary outcome 1: tobacco use	Tobacco use status	% of enrolled NCD patients who were using tobacco at enrollment who are nonusers or who have reduced by > 50% their tobacco intake, at completion of the study period
	Secondary outcome 2: alcohol use	Alcohol use status	% of enrolled NCD patients who were alcohol drinkers at enrollment who are nondrinkers or who have reduced by > 50% alcohol intake, at completion of the study period
Specific Aim 2: implementation	Reach	Home visit coverage	% of enrolled NCD patients having a CHW home visit, measured monthly
		Clinic visit coverage	% of enrolled NCD patients having an MLP visit at the clinic, measured monthly according to the patients indicated to be seen that month based on protocol-based guidelines
		Demographic, geographic barriers and facilitators	% of enrolled NCD patients whose CHW has GPS-mapped their households, describing barriers/facilitators to individuals’ access, and identifying contributors to variation/inequities
		Loss to follow-up	% of patients, stratified by demographic data and NCD conditions, who are lost to follow-up after enrollment
		Monthly patient touch-points	Number of monthly per-patient touch-points, including interactions by both MLPs and CHWs
	Efficacy	Evidence-based hypertension management	% of enrolled hypertension patients in accordance with evidence-based recommendations, as prescribed by clinical algorithms, assessed quarterly by EHR audits
		Evidence-based diabetes management	% of enrolled diabetes patients in accordance with evidence-based recommendations, as prescribed by clinical algorithms, assessed quarterly by EHR audits
		Evidence-based COPD management	% of enrolled COPD patients in accordance with evidence-based recommendations, as prescribed by clinical algorithms, assessed quarterly by EHR audits
	Adoption	Village-cluster adoption	% of intended village clusters receiving intervention
		Timely adoption	% of intended village clusters rolling-out intervention within 3 months of schedule, according to local governance decisions to roll-out the intervention
		CHW adoption	% of CHWs trained in intervention implementation within the first 6 months % of trained CHWs retained in their positions at completion of the study period
		MLP adoption	% of MLPs trained in intervention implementation % of trained MLPs retained in their positions at completion of the study period
	Implementation	Care integration	% of all NCD patients enrolled at the facilities seen by CHWs at home within the first month
		CHW supervision model	% of scheduled CHW supervision field visits completed, stratified by CHN and district, measured quarterly % of scheduled quarterly data review meetings held with CHWs and CHNs, measured quarterly
		CHW home visit fidelity	% of enrolled NCD patients with 100% of algorithm-indicated home visits received % of topics included at each session as dictated by the condition-specific algorithms, assessed during the CHW supervision field visits by CHNs, measured quarterly
		Referrals	% of patients appropriately referred to MLP care as indicated by the clinical algorithms, assessed during the CHW supervision field visits by CHNs, measured quarterly % of patients referred by CHWs seen by MLPs within the prescribed time window according to the clinical algorithms (e.g., 24 h, 72 h, 1 week), measured quarterly
		MLP supervision model	% of enrolled NCD patients appropriately referred to see a physician by MLPs as indicated by the clinical algorithms, assessed during monthly physician supervision sessions, measured quarterly
		MLP visit fidelity	% of enrolled NCD patients with 100% of algorithm-indicated facility visits received, assessed during monthly physician supervision sessions, measured quarterly % of diagnostic, treatment, and counseling topics included at each session as dictated by the condition-specific algorithms, assessed during monthly physician supervision sessions, measured quarterly
		Implementation challenges	Exploratory and hypothesis-generating as revealed through FGDs and KIIs with CHWs, CHNs, MLPs, physicians, patients, and other relevant community stakeholders
	Maintenance	Total intervention cost	Cost of each intervention component and total costs using the Joint Learning Network costing methodology
		Intervention initiation costs	% breakdown of initial (one-time) costs for intervention (training, equipment, etc.)
		Intervention maintenance costs	% breakdown of maintenance (recurring) costs (ongoing training, personnel, materials, and other)
		Facility vs. community costs	% of costs of healthcare divided between facility level and community level
		Geographic cost variation	Characterization of variance in costs between village clusters and districts within the intervention catchment area
		Out-of-pocket patient costs	% costs of healthcare divided between facility level and community level
		Integrated intervention cost-effectiveness analysis	Pre/post-intervention marginal effectiveness for primary outcomes
		Cost per unit	Intervention cost per enrolled patient Intervention cost per capita Projected cost to scale intervention nationally, based on known incidence and prevalence of each condition

*CHN* community health nurse, *CHW* community health worker, *COPD* chronic obstructive pulmonary disease, *EHR* electronic health record, *FGD* focus group discussion, *GPS* Global Positioning System, *KII* key-informant interview, *MLP* mid-level practitioner, *NCD* Noncommunicable disease, *RE-AIM* Reach, Effectiveness, Adoption, Implementation, Maintenance

**Table 2:** Clinical definitions of “at-goal” status for each intervention condition

Noncommunicable disease	Management metric	“At-goal” definition
Type II diabetes mellitus	Hemoglobin A1c OR fasting blood sugar	Hemoglobin A1c < 7.5 OR fasting blood sugar < 130 mg/dl^a^
Hypertension	Blood pressure	Blood pressure < 130/80 mmHg or patient-tailored goal per risk stratification^b^
Chronic obstructive pulmonary disease	Exacerbation status	≤ 1/3 Anthonisen criteria^c^

^a^The 2018 American Diabetes Association guidelines [82] call for a goal A1c < 7% for most patients or A1c < 8% in “patients with a history of severe hypoglycemia, limited life expectancy, advanced microvascular or macrovascular complications, extensive comorbid conditions, or long-standing diabetes in whom the goal is difficult to achieve despite diabetes self-management education, appropriate glucose monitoring, and effective doses of multiple glucose-lowering agents including insulin.” For our intervention, we established 7.5% as our goal to pragmatically accommodate both populations

^b^Based on the 2017 American College of Cardiology and American Heart Association guidelines [83], we established < 130/80 mmHg as a default goal, with patient-tailored goals for select patients (≥ 65 years of age, multiple comorbidities, limited life expectancy, clinical judgment, patient preference)

^c^The 2017 update to the GOLD guidelines [84] define chronic obstructive pulmonary disease exacerbation as an “acute worsening of respiratory symptoms that results in additional therapy.” We used the Anthonisen criteria of worsening sputum volume, sputum purulence, and increased dyspnea to define the “worsening of respiratory symptoms” specified in the GOLD guidelines. We established a threshold of no more than one Anthonisen criterion as a pragmatic tool for determining clinical status

Secondary outcomes for Specific Aim 1 will include the following. We will assess the individual “at-goal” rates per condition. We will assess the persistence of the intervention for the subset of patients for whom we have the data (i.e., those enrolled within 12 months of the start of the study) on their 24 months’ outcome. Additionally, we will examine the tobacco and alcohol status of enrolled patients, specifically focusing on the proportion of patients who were tobacco users and/or alcohol drinkers at the time of enrollment who have stopped all tobacco and/or alcohol intake, or reduced their intake by > 50%, by the completion of the study period (Table 1).

For Specific Aim 2, the RE-AIM framework will be utilized to assess the implementation of the study intervention, with RE-AIM metrics as presented in Table 1.

### Sampling strategy and power calculations

We will use exhaustive convenience sampling to screen all eligible patients seen across two facilities over a 12-month period into the analysis cohort. Based on historical formative data of patient volume seen at these two facilities, and accounting for an expected 30% attrition rate, we conservatively expect that at least 1000 patients will be eligible for enrollment into the cohort.

With this conservative number of 1000 as our expected sample size based on this convenience sampling, we conducted power calculations to determine the statistical power to detect a change in the “at-goal” status. We calculate power based on a simplified design to compare paired proportions using a two-sided McNemar’s test with an 0.05 type I error (α) level. The primary outcome is the proportion of patients who achieve their NCD control target (“at-goal status”) after 12 months of being engaged in care. We used SAS version 9.4 (Cary, NC, USA) to estimate power to detect a 5% difference between discordant proportions; that is, proportions of patients whose “at-goal” status changed from “not at-goal” at baseline to “at-goal” at follow-up, and vice versa, in multiple scenarios where the total proportion of discordant patients ranged from 10 to 40% of all patients. Based on these assumptions, our power to detect a 5% difference in the discordant pairs is 69%, when the total discordant proportion is 40%, and the power is 99% when 10% of all patients are discordant.

### Data collection

#### Quantitative data

Quantitative data for patient outcomes will be extracted from the facility-based EHR and the CHW’s mobile-phone application (Additional file 5), and will be used to assess Specific Aims 1 and 2. (Table 1) All implementation-related data for evaluating the performance of MLPs and CHWs (Table 1) will be collected by the responsible MLP and CHW supervisors in digitized checklists within the EHR and mobile-phone application. Access to protected health information will be controlled and defined by user access groups according to clinician status. Data to be analyzed will be extracted via secure data queries from the EHR system in an aggregate, partially de-identified form, with external researchers signing a data-sharing and use agreement prior to analysis. Cleaned, de-identified datasets will be made publicly available via a data repository.

Costing data for the intervention will be collected utilizing a “top-down” method, as described by the Joint Learning Network [85]. This method will document direct and indirect costs associated with the NCD care delivery intervention described here, and related administrative functions (including planning and administration; training; supervision and monitoring and evaluation; data management; and continuous surveillance) will be disaggregated. Full methodology of direct and indirect costs is provided by the Joint Learning Network [85], and will be utilized for this study. For the purposes of this pragmatic study, this methodology will be appropriate to estimate the additional marginal costs of the intervention (rather than cost-savings or secondary cost implications) as compared to general standard of care.

#### Qualitative data

Qualitative data will be used for Specific Aim 2 (Table 1). Staff members, patients, community leaders, and government officials will be approached for KIIs and FGDs. Purposive sampling will be used, aiming to maximize heterogeneity across sex, socioeconomic position, healthcare issues, geographic location, age, caste class, and other attributes. For each group, five key informant interviews will be conducted at each time point, as described in the following. One focus group discussion per group will be conducted at each time point.

KII and FGD guides will be developed in advance, and will vary across the study period, exploring specific topics of concern. A locally validated, seven-domain framework of healthcare delivery analysis will be used to inform data collection [86]. These seven domains include health service operations, supply chains, equipment, personnel, outreach, societal factors, and structural factors. Qualitative data collection will focus on these areas to assess the implementation of the intervention.

FGDs and KIIs will occur prior to the initiation of the intervention, and at intervals of 6 months throughout the study period, to assess ongoing implementation status. All sessions will be conducted in Nepali. All qualitative data will be stored on a Research Electronic Data Capture (REDCap) database [87]. REDCap user access will be defined so that researchers only have access to de-identified study data. Any paper copies of data forms will be stored in locked cabinets inside locked rooms at district facilities. Once all data are fully transcribed and validated for quality, all paper copies will be destroyed. REDCap data will be deleted 12 months after the completion of the study period.

### Data analysis

#### Analysis for Specific Aim 1: effectiveness

In order to assess the effectiveness of the intervention, as already described, the primary outcome will utilize disease-specific “at-goal” metrics for each of the three study diseases: hypertension, type II diabetes, and COPD. We hypothesize that the integrated intervention will lead to a 10% increase in the “at-goal” status of the combined disease cohorts, over a 12-month follow-up period.

We will use conditional multivariable logistic regression to assess patient outcomes at 12 months of follow-up, adjusting for potential confounding and/or effect modification by patients’ demographics (including age, sex, caste), municipality, district, mean distance to the hospital, and engagement in care (defined as number of facility-based and community-based encounters). We additionally hypothesize a 10% improvement in the status of each of the two secondary outcomes: tobacco and alcohol use, as measured by patient-reported outcomes presented in Table 1.

As a secondary analysis for Specific Aim 1, namely the time-varying nature of the outcomes, we will assess the longitudinal effect of the intervention, as measured in 3-monthly intervals, throughout the study period, compared to baseline statistics at the time of each village-cluster enrollment. Variables will be considered either nominal or continuous (linear effect) predictors, and the generalized linear model framework will be used to estimate effect of time-varying repeated-measure intervention implementation over the several steps of the wedged design. Differential impact from time of intervention will be evaluated with test of month × intervention interaction. Models will be fit using generalized estimating equations, for example, using SAS Proc Genmod, to calculate valid standard errors in the presence of repeated measures over time and possibly correlated outcomes at the municipality level. Assumptions of overdispersion or underdispersion will be examined closely, and an estimated scale parameter or negative binomial models will be used as needed.

#### Analysis for Specific Aim 2: RE-AIM implementation framework

In this mixed-methods study, Specific Aim 2 will be assessed using the RE-AIM framework for implementation trials [80]. A full list of metrics, separated by each domain of the RE-AIM framework, is presented in Table 1. Additional details regarding the supervision and audit structure for MLPs and CHWs can be found in Additional files 1 and 2.

For the maintenance of the intervention, we will assess the costs of the intervention, using the Joint Learning Network methodology [85]. Cost data will be analyzed and presented (Table 1) to help program planners and policy-makers understand the implications for possible scale of a similar intervention by the government or other entity in the future.

For quantitative data within Specific Aim 2, a similar methodology of generalized estimating equations, as already described in Analysis for Specific Aim 1: effectiveness, will be applied. Data will be assessed at 3-month intervals.

For qualitative data within Specific Aim 2, analysis will be ongoing and iterative, so as to continually inform further qualitative data collection, focusing on timely and relevant implementation issues. Data from KIIs and FGDs will transcribed and coded using grounded theory methodology [88, 89]. NVivo software will be used for qualitative data analysis [90].

## Discussion

### Ethical approval and consent

This study has been approved by the Ethical Review Board of the Nepal Health Research Council (#177/2018). Within the study, all patients will provide verbal informed consent to have their de-identified data analyzed and published. Care provision will be unrelated to consent, and there will be no difference in care provision based on consent status. Verbal informed consent will also be provided by all KII and FGD participants. No incentives will be provided to study participants, to avoid any conflict of interest or coercion to participate. Protocol modifications will be promptly communicated to the IRB and on the trial registry website by members of the research study team.

### Safety considerations

There are minimal risks posed to patients, staff, or other key informants. The predominant risk is disclosure of protected health information, and/or qualitative data from KIIs or FGDs. All patient information will be stored on secure databases, and data access privileges will be heavily restricted. Unless otherwise deemed necessary for a specific analysis, all analyses will be conducted using a limited dataset. Qualitative data will be stored and protected as already described.

### Data sharing

All de-identified data from this study will be made publicly available for other researchers to analyze at their discretion in the future, to further this field of research. De-identified summaries of qualitative data will be made available as well.

### Dissemination plan

Domestically within Nepal, 6-monthly update meetings will be held between researchers and the Ministry of Health and Population to review ongoing results. When completed, results of the study will be presented at the annual National Summit of Health and Population Scientists, organized by the Nepal Health Research Council, and at other relevant international conferences. Peer-reviewed publications will be drafted for international dissemination.

## Trial status

At the time of manuscript submission, the study is currently not yet recruiting participants. Participant enrollment is anticipated to commence in February 2020 and is planned to continue for 1 year. Intervention deployment took place between July 2018 and will conclude in January 2020. This is study protocol version 1.2 and the version date is December 6, 2019.

## Supplementary information

**Additional file 1.** Detailed description of intervention.

**Additional file 2.** Community and facility-based supervision structures.

**Additional file 3.** Informed consent form.

**Additional file 4.** SPIRIT 2013 Checklist: Recommended items to address in a clinical trial protocol and related documents.

**Additional file 5.** Interview guides and data collection tools.

## Data Availability

The datasets supporting the conclusions of the study will be made publicly available in de-identified form upon conclusion of the study. The final trial dataset (in limited identifier format) will be accessible to researchers at the research performance site in Nepal—Nyaya Health Nepal—and co-investigators assisting with data analysis. Participating research institutions will enter into data-sharing agreements (namely between the research performance site in Nepal—Nyaya Health Nepal—and any foreign institutions where investigators are assisting with data analysis) covering terms of access to specific limited datasets; provisions for storing, sharing, and using data; and methods for securing data transfer.

## Abbreviations

CDS: Clinical decision support
CHN: Community health nurse
CHW: Community health worker
COPD: Chronic obstructive pulmonary disease
EHR: Electronic health record
FGD: Focus group discussion
HEARTS: Healthy-lifestyle counselling. Evidence-based treatment protocols. Access to essential medicines and technology. Risk based charts. Team-based care. Systems for monitoring
KII: Key-informant interview
LMIC: Low and middle-income country
MI: Motivational interviewing
MLP: Mid-level practitioner
NCD: Noncommunicable disease
RE-AIM: Reach, Effectiveness, Adoption, Implementation, Maintenance
REDCap: Research Electronic Data Capture
WHO: World Health Organization
WHO-PEN: WHO Package of Essential Noncommunicable Disease Interventions
