# Supplementary material for: An integrated intervention for chronic care management in rural Nepal: protocol of a type 2 hybrid effectiveness-implementation study
Source: Trials. 2020 Jan 29;21:119. doi: 10.1186/s13063-020-4063-3 (PMC6990567; doi:10.1186/s13063-020-4063-3)
Supplement: Supplementary file 1 — Additional file 1. Detailed description of intervention. [file 13063_2020_4063_MOESM1_ESM.docx]

**Supplemental File 1:** **Detailed description of intervention**

*Introduction*

In this supplemental file, we provide a detailed description of the study intervention. In addition to the government’s planned World Health Organization Package of essential non-communicable disease interventions (WHO-PEN) program roll-out, this study will include three evidence-based components: 1) non-communicable disease (NCD) care integration using mid-level practitioners (MLPs) and community healthcare workers (CHWs); 2) clinical decision support (CDS) tools to ensure quality care in accordance with best practices; and 3) training and supervision of MLPs to provide motivational interviewing (MI) techniques for modifiable risk factor optimization, with a specific focus on tobacco and alcohol use.

*Non-communicable disease care integration using MLPs and CHWs*

The NCD care management intervention focuses on integrating care delivery between the facility and community level, using MLPs at the facilities and CHWs in the communities. These settings are further linked by the EHRs and mobile phone applications, which are described in greater detail below.

In Nepal, there is already a precedent of MLPs providing the majority of outpatient care, [1] and CHWs called Female Community Health Volunteers, providing community-based service delivery,[2] but these systems have not been integrated optimally for NCD management. Our intervention strengthens the existing MLP cadre at the facility by recruiting additional staff members, providing intensive NCD-focused training on a regular basis with daily continuing medical education programs, and ensuring quality supervision by a cadre of Bachelor of Medicine, Bachelor of Surgery (MBBS) level staff physicians and senior general practitioner physicians.

The MLPs in the intervention are from the Nepali cadre of health assistants (HAs), who have three years post-secondary healthcare education. In the intervention, the primary care NCD services are delivered predominantly by HAs. HAs are responsible for algorithmic, diagnostic and treatment services driven by the WHO-PEN, in addition to health education for patients, which is described in greater detail below.

The HAs in this intervention are overseen at the facilities by a cadre of MBBS-trained staff physicians, who are, in turn, overseen by several general practitioner residency-trained physicians (called “MD-GP” physicians in the local setting). The HAs receive daily, on-going supportive supervision and continuing medical education sessions, targeted at the MLP-level of education, facilitated by the MBBS and MD-GP physicians (Supplemental File 2).

In the community, we have developed a cadre of CHWs, which has been previously described,[3, 4] The CHWs in our intervention are a novel cadre of workers in Nepal, based on global best practices and the growing body of evidence surrounding CHW program development.[5] This cadre is notably different from the pre-existing Female Community Health Volunteers, and has been developed over the past ten years as part of the public-private partnership between *Nyaya Health Nepal* and the Ministry of Health and Population. These CHWs are all female, from the communities they serve, with a minimum of tenth grade (“School Leaving Certificate”) education. They are all fully-employed and salaried, integrated into the organizational management structure. To ensure accountability and quality assurance, they are overseen by Community Health Nurses (CHNs), who are, in turn, overseen by Community Healthcare Program Associates (CHPAs). CHNs are fully-qualified nurses from the Nepali staff nurse or auxiliary nurse midwife cadres, having received either three years or eighteen months post-secondary education, respectively. All CHNs are registered with the Nepal Medical Council as licensed practitioners. The CHPAs oversee the CHNs and are, in turn, overseen by one district-level Community Healthcare District Manager. This network of CHWs has been described in greater detail previously.[3, 6, 7]

The CHWs all receive an initial healthcare-focused training at the time of hiring, and then on-going weekly continuing education and supportive supervision. The CHWs are supervised by CHNs one to two times per month, during the CHWs’ routine home-visits for patients. The system is designed to provide continual supportive supervision and improvement-focused feedback, during the course of their regular daily work responsibilities. In addition to these supervision visits, the CHWs attend at least twice-monthly meetings at central offices at their service hub, which vary in location, but are mostly based at village healthcare facilities throughout the districts. During these meetings, they have direct one-to-one review of their patient records with CHNs, and on-going training. The CHPAs oversee these meetings, providing structured teaching and mentorship to the CHNs and CHWs, reviewing the patient-related data, and identifying areas for targeted improvement.

At the community level, for NCD patients specifically, CHWs are currently trained to provide post-facility visit follow-up, reviewing medication adherence, side effect screening, and general NCD health education as provided in the PEN packages. They visit each NCD patient once monthly, and on an as-needed basis when instructed by the MLPs or requested by the patients themselves.

*Clinical decision support tools to ensure quality care in accordance with best practices*

To address continuity of care between visits, we have developed a digital network linking facility-based and community-based visits. At the facilities, MLPs utilize an EHR system that has been described previously, designed for resource-limited settings.[8] In the community, all CHWs are equipped with an Android-based mobile phone, with the CommCare application installed. By linking patient records across facility and community settings, both MLPs and CHWs have access to all patient data, decreasing the discontinuity and fragmentation associated with unlinked visits across different settings and time.

Furthermore, these digital tools enable the use of clinical decision support for MLPs and CHWs. Well-described globally, variances in quality of care[9] – the “know-do gap” – between providers and facilities, are a significant barrier to quality NCD provision at scale. Our intervention employs protocol-based algorithmic care[10-12] at both facility and community visits, designed to optimize consistent quality from visit to visit, in line with evidence-based practices. These clinical decision support tools are integrated into the EHR at facilities and within the CommCare application used by CHWs in the communities.

At the facilities, clinical decision support features guide MLPs through templated condition-specific protocols. While this intervention focuses on a limited set of specific conditions (hypertension, type II diabetes, and chronic obstructive pulmonary disease (COPD)), the EHR clinical decision support tools are designed to encompass a large set of common conditions relative to the local setting. For NCDs, these algorithms are based on PEN protocols.

In the community, CHWs use the CommCare application, an open-source mobile phone platform for healthcare workers, which has been used in over 50 countries globally, and has been studied in 39 peer-reviewed publications.[13] CommCare and other clinical decision support tools are well-equipped to provide guidance for healthcare workers providing care for which clear algorithms exist, such as the PEN protocols. CommCare includes capabilities for data collection, decision support, task reminders, and counseling tools. The mobile application can be accessed and used in the community offline and the data are uploaded at a later time when online services are accessible.

In this intervention, we have customized the CommCare application to include NCD condition-specific modules based on PEN protocols. The CHWs utilize these condition-specific templated modules during the course of their home visits, to assure that their NCD community-based services are in line with evidence-based practices. Data from the CHWs’ individual CommCare accounts are analyzed routinely by CHNs and CHPAs to provide improvement-focused feedback during their in-person meetings, as described above.

*Motivational interviewing techniques for modifiable risk factor optimization*

To provide comprehensive NCD care management, including both preventative and curative services, individual-level risk factor modification counseling is a critical aspect of the intervention. While many of our patients’ health issues are related to social and environmental factors outside the scope of healthcare alone,[14, 15] our intervention prioritizes individual-level counseling using MI techniques to improve our patients’ circumstances to prevent disease and, when present, the progression of these NCDs. This counseling is informed by WHO-PEN “brief intervention” guidance,[16] in addition to MI techniques. As a component of all facility visits, patients spend time (usually between 5-10 minutes per visit) with a dedicated NCD-specific MLP counselor. Using a standardized template, MLPs provide counseling and education about modifiable risk factors such as tobacco, alcohol, diet and nutrition; medication adherence and treatment side effects; planned follow-up needs; and other condition-specific issues. In the community, CHWs use similar protocols to discuss risk factors, medication adherence, and follow-up.

**References**

1. Knoble, S.J. and Bhusal, M.R., Electronic diagnostic algorithms to assist mid-level health care workers in Nepal: A mixed-method exploratory study*.* *Int J Med Informatics*, 2015. **84**(5): 334-340.

2. Neupane, D., McLachlan, C.S., Mishra, S.R., et al., Effectiveness of a lifestyle intervention led by female community health volunteers versus usual care in blood pressure reduction (COBIN): an open-label, cluster-randomised trial*.* *Lancet Global Health*, 2018. **6**(1): e66-e73.

3. Citrin, D., Thapa, P., Nirola, I., et al., Developing and deploying a community healthcare worker-driven, digitally- enabled integrated care system for municipalities in rural Nepal*.* *Healthcare*, 2018.

4. Ballard, M. and Schwarz, R., Employing practitioner expertise in optimizing community healthcare systems*.* *Healthcare J Delivery Sci Innov*, 2018.

5. Ballard, M., Schwarz, R., Johnson, A., et al. Practitioner Expertise to Optimize Community Health Systems: Harnessing Operational Insight. 2018; Available from: <https://chwimpact.org>.

6. Schwarz, D., Sharma, R., Bashyal, C., et al., Strengthening Nepal's Female Community Health Volunteer network: a qualitative study of experiences at two years*.* *BMC Health Serv Res*, 2014. **14**: 473.

7. Maru, S., Nirola, I., Thapa, A., et al., An integrated community health worker intervention in rural Nepal: a type 2 hybrid effectiveness-implementation study protocol*.* *Implementation Science*, 2018. **13**(1): 53.

8. Raut, A., Yarbrough, C., Singh, V., et al., Design and implementation of an affordable, public sector electronic medical record in rural Nepal*.* *J Innov Health Inform*, 2017. **24**(2): 10.

9. Das, J., Woskie, L., Rajbhandari, R., et al., Rethinking assumptions about delivery of healthcare: implications for universal health coverage*.* *BMJ*, 2018. **361**: k1716.

10. World Health Organization, Implementation tools: package of essential noncommunicable (PEN) disease interventions for primary health care in low-resource settings. 2013, World Health Organization: Luxembourg. 210.

11. Government of Nepal, M.o.H., Department of Health Services. National Health Training Center. 2014 [cited 2018 20 Feb 2018]; Available from: <http://dohs.gov.np/centers/national-health-training-center/>.

12. Nick Simons Institute. Mid-Level Practicum. 2018 [cited 2018 20 Feb 2018]; Available from: <http://nsi.edu.np/en/page/programs/training/mid_level_practicum>.

13. Chatfield, A., Javetski, G., and Lesh, N., Commcare evidence base*.* *Dimagi web site*, 2013.

14. Clark, J., Medicalization of global health 3: the medicalization of the non-communicable diseases agenda*.* *Global Health Action*, 2014. **7**(1): 24002.

15. Aryal, K.K., Mehata, S., Neupane, S., et al., The burden and determinants of non communicable diseases risk factors in Nepal: findings from a nationwide STEPS survey*.* *PloS One*, 2015. **10**(8): e0134834.

16. Package of Essential Noncommunicable (PEN) disease and healthy lifestyle interventions: Training modules for primary health care workers, 2018, R.O.f.S.-E.A. World Health Organization: New Delhi.
