# Supplementary figures and images for: An integrated intervention for chronic care management in rural Nepal: protocol of a type 2 hybrid effectiveness-implementation study
Source: Trials. 2020 Jan 29;21:119. doi: 10.1186/s13063-020-4063-3 (PMC6990567; doi:10.1186/s13063-020-4063-3)

# Community and facility-based supervision structures

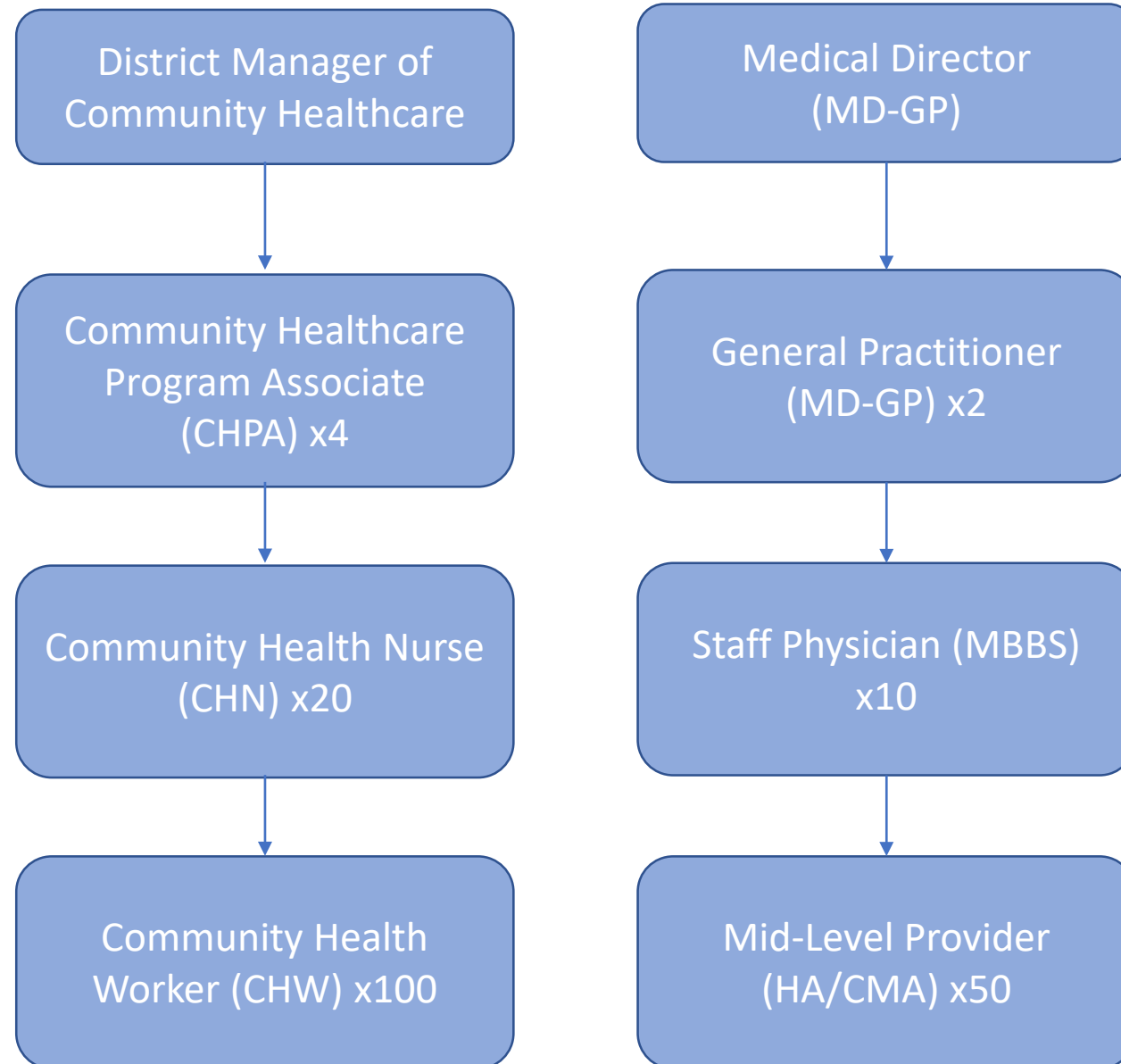

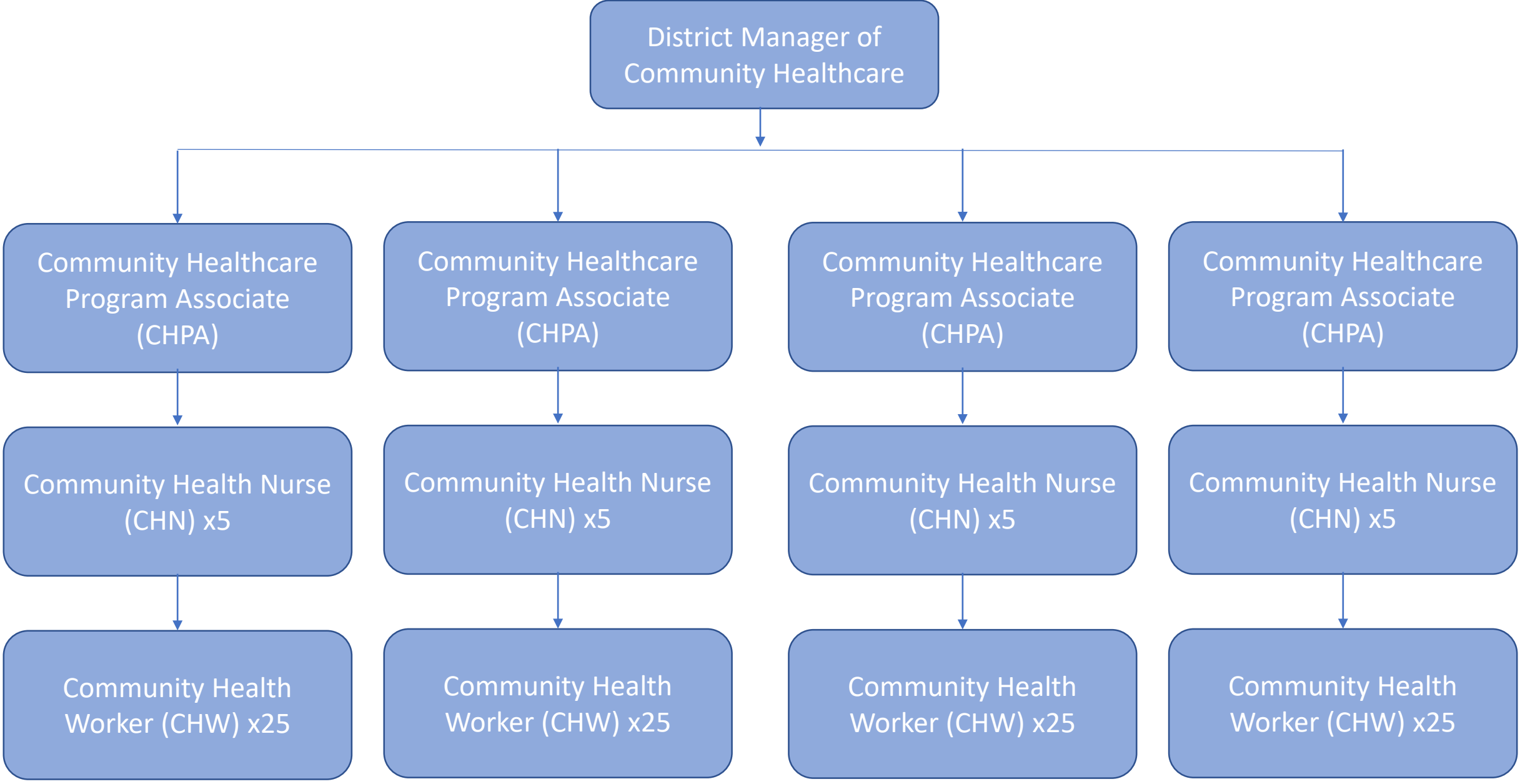

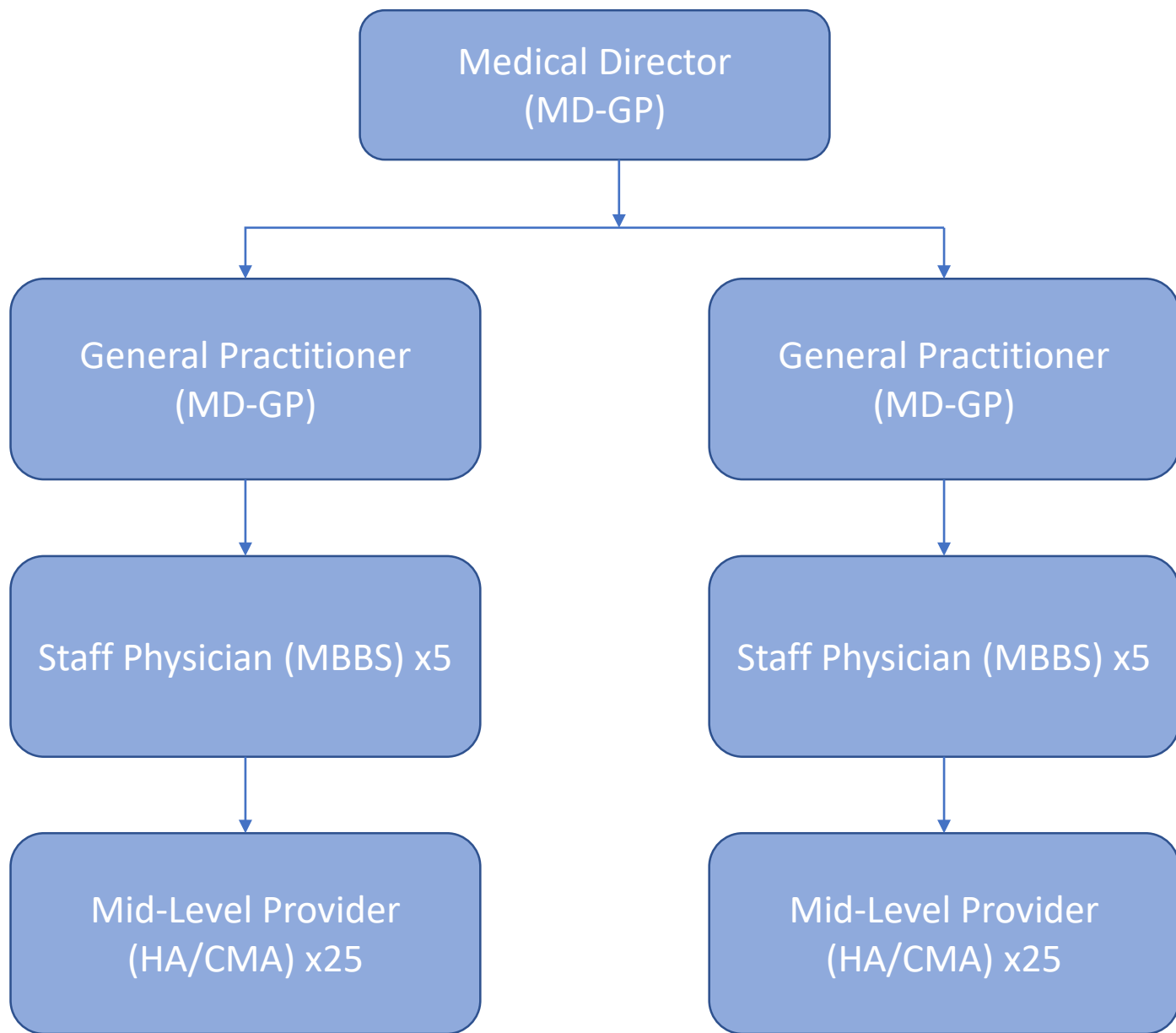

Supplement: Supplementary file 2 — Additional file 2. Community and facility-based supervision structures. [file 13063_2020_4063_MOESM2_ESM.pdf]
