# Supplementary material for: An integrated intervention for chronic care management in rural Nepal: protocol of a type 2 hybrid effectiveness-implementation study
Source: Trials. 2020 Jan 29;21:119. doi: 10.1186/s13063-020-4063-3 (PMC6990567; doi:10.1186/s13063-020-4063-3)
Supplement: Supplementary file 3 — Additional file 3. Informed consent form. [file 13063_2020_4063_MOESM3_ESM.pdf]

Implementing a modified WHO Package of Essential Non-communicable disease interventions protocol (PEN PLUS) by non-physician healthcare workers in Achham and Dolakha: a combined acceptability/feasibility and type 2 hybrid-effectiveness implementation research study

INFORMED CONSENT – Patient Participants

Investigators:

Duncan Maru, MD, PhD	Santosh KD
Chief Strategy Officer	Medical Director-Bayalpata Hospital
Nyaya Health Nepal - Possible	Nyaya Health Nepal-Possible
CONTACT: +1-6175213381	CONTACT: +977-9856036194

Investigator's Statement

You are invited to participate in a study conducted by the Ministry of Health and Nyaya Health Nepal-Possible. We hope to learn more on how to improve the care for patients with non-communicable diseases by implementing a modified WHO protocol called 'Package of essential non-communicable disease interventions – PLUS'. You were selected as a possible participant in this study because you are receiving care for one or more non-communicable diseases (diabetes, COPD/asthma, hypertension) which we are studying and you live in the Nyaya Health Nepal catchment area population.

The purpose of this consent script is to give you the information you need to help you decide whether or not to be in the study. When all your questions have been answered, you can decide if you want to be in the study or not. This process is called "informed consent."

Procedures

If you agree, your participation will involve the following:

- Household healthcare visits: you will receive routine and follow-up clinical care from CHWs serving your community as determined by your medical record and healthcare needs; each visit with a provider will vary in length depending on your healthcare needs.
- Health outcome analysis: we will look at your medical records and analyze data on your health outcomes: (including: health outcomes, geographic location, caste/ethnicity, and medical history); no direct involvement beyond receiving routine clinical care. *More details on data to be analyzed can be provided; see protocol 14.4 Research Aims/17. Data Analysis plan.
- Focus group discussion: you may be invited to attend a focus group discussion with other participants from your community (1 time); the discussion will take between 30-60 minutes. Your answers will be recorded and the interviewer(s) will take field notes. *Research staff may contact you to clarify your responses.
- Key-informant interview: you may be invited to attend an interview, one-on-one with a research staff member. The interview may last between 30-60 minutes. Your answers will be recorded and the interviewer(s) will take field notes. *Research staff may contact you to clarify your responses.

Risks and Benefits of the Study

You may not directly benefit from taking part in this study. However, we hope the program will provide you with direct clinical care for you. We also hope the results of the study will improve delivery of non-communicable disease care in Achham, Dolakha, and throughout Nepal.

Risks of participation include breach of privacy and disclosure of any personal information.

Other Information
Any information that is obtained in connection with this study and that can be identified with you will remain confidential. When we share information from this study, it will not be connected with your name or family.

We may also wish to publish quotes arising from your focus group discussions and/or interview. These quotes will not contain any identifiable information about you or your child (including: name, age, sex, caste/ethnicity, subject/medical record identifiers, contact information, geographical address, and detailed information about your medical history). We may wish to use these quotes in one or more of the following publications:
- Journal article; open-access publication shared with research community
- Lay-press/newspaper article; either national or international news source
- Conference/symposium; poster, individual/group panel presentation
- Research student thesis/dissertation

Your decision to participate in this study and your responses during focus group discussions/interviews will not affect your care at (Bayalpata Hospital/Charikot Primary Health Center/CHWs in your community) now or in the future. If you decide to participate in the study, you can change your mind and withdraw your consent at any time during or after any of the procedures.

This research has been approved by the following bodies: Brigham and Women's Hospital, and Nepal Health Research Council. If you have any questions, please ask us. If you have any additional questions later, contact Santosh Dhungana, Medical Director-Bayalpata Hospital, +977-9856036194, who will be happy to answer them.

You are making a decision whether or not to participate. Your verbal confirmation indicates that you have decided to participate, having been read and explained the information provided above.

[Please ask for participant to explain the study, procedures, risks/benefits to confirm their understanding. Encourage questions and provide any additional explanations.]

Date: ______________________

Verbal Consent (circle one): YES NO

[Indicate in CommCare/Bahmni [form field X/Y] their consent to participate in research study.]

Implementing a modified WHO Package of Essential Non-communicable disease interventions protocol (PEN PLUS) by non-physician healthcare workers in Achham and Dolakha: a combined acceptability/feasibility and type 2 hybrid-effectiveness implementation research study

INFORMED CONSENT – Staff Participants

Investigators:

Duncan Maru, MD, PhD	Santosh KD
Chief Strategy Officer	Medical Director-Bayalpata Hospital
Nyaya Health Nepal - Possible	Nyaya Health Nepal-Possible
CONTACT: +1-6175213381	CONTACT: +977-9856036194

Investigator's Statement

You are invited to participate in a study conducted by the Ministry of Health and Nyaya Health Nepal-Possible. We hope to learn more on how to improve the care for patients with non-communicable diseases by implementing a modified WHO protocol called 'Package of essential non-communicable disease interventions – PLUS'. You were selected as a possible participant in this study because you are involved in implementing the intervention (e.g. either CHW, NPHW, physician, other clinical/non-clinical team member).

The purpose of this consent script is to give you the information you need to help you decide whether or not to be in the study. When all your questions have been answered, you can decide if you want to be in the study or not. This process is called "informed consent."

Procedures

If you agree, your participation will involve the following:

- Focus group discussion: you may be invited to attend a focus group discussion with other staff members involved in the intervention (1 time); the discussion will take between 30-60 minutes. Your answers will be recorded and the interviewer(s) will take field notes. *Research staff may contact you to clarify your responses.
- Key-informant interview: you may be invited to attend an interview, one-on-one with a research staff member, following the focus group discussion (1 time); interview may last between 30-60 minutes. Your answers will be recorded and the interviewer(s) will take field notes. *Research staff may contact you to clarify your responses.

Risks and Benefits of the Study

You may not directly benefit from taking part in this study. We do hope that the results of the study will improve delivery of reproductive, maternal, newborn and child health services in Achham, Dolakha, and throughout Nepal.

Risks of participation include breach of privacy and disclosure of any personal information.

Other Information

Any information that is obtained in connection with this study and that can be identified with you will remain confidential. When we share information from this study, it will not be connected with your name or family.

We may also wish to publish quotes arising from your focus group discussions and/or interview. These quotes will not contain any identifiable information about you (including: name, age, sex, caste/ethnicity, subject/medical record identifiers, contact information, geographical address, and detailed information about your medical history). We may wish to use these quotes in one or more of the following publications:

- Journal article; open-access publication shared with research community
- Lay-press/newspaper article; either national or international news source
- Conference/symposium; poster, individual/group panel presentation
- Research student thesis/dissertation

Your decision to participate in this study and your responses during focus group discussions/interviews will not affect your employment or institutional affiliation (with the Ministry of Health and/or Nyaya Health Nepal-Possible) now or in the future. If you decide to participate in the study, you can change your mind and withdraw your consent at any time during or after any of the procedures.

Participant Unable to Read/Write:
If you are unable to read/write, you are entitled to have a witness present to assist you with the informed consent process. A witness can help you interpret the information being presented to you and can assist you with making an appropriate decision.

[If participant is unable to read/write, please offer them to have a witness present during the consent process.]

This research has been approved by the following bodies: Brigham and Women's Hospital, and Nepal Health Research Council. If you have any questions, please ask us. If you have any additional questions later, contact Santosh Dhungana, Medical Director-Bayalpata Hospital, +977-9856036194, who will be happy to answer them.

You are making a decision whether or not to participate. Your verbal confirmation indicates that you have decided to participate, having been read and explained the information provided above.

[Please ask for participant to explain the study, procedures, risks/benefits to confirm their understanding. Encourage questions and provide any additional explanations.]

Date: ______________________

Verbal Consent (circle one): YES NO

**[If YES -> Proceed for inclusion in Focus Group Discussion/Key-Informant Interview.
If NO -> Remove from Focus Group Discussion/Key-Informant Interview participant list.]**

**[Written Informed Consent using witness: name: ______________________________________
sig: ____]**
