# Supplementary material for: An integrated intervention for chronic care management in rural Nepal: protocol of a type 2 hybrid effectiveness-implementation study
Source: Trials. 2020 Jan 29;21:119. doi: 10.1186/s13063-020-4063-3 (PMC6990567; doi:10.1186/s13063-020-4063-3)
Supplement: Supplementary file 4 — Additional file 4. SPIRIT 2013 Checklist: Recommended items to address in a clinical trial protocol and related documents. [file 13063_2020_4063_MOESM4_ESM.docx]

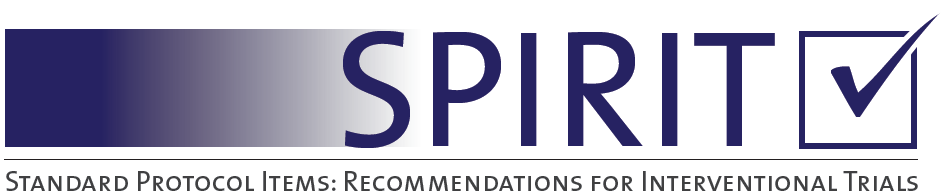


SPIRIT 2013 Checklist: Recommended items to address in a clinical trial protocol and related documents*

| Section/item | Item No | Description | | Addressed on page number |
| --- | --- | --- | --- | --- |
| **Administrative information** | | | |  |
| Title | 1 | An integrated intervention for chronic care management in rural Nepal: a type 2 hybrid effectiveness-implementation study | | 1 |
| Trial registration | 2a | Registration in ClinicalTrials.gov is currently pending. | | 4 |
|  | 2b | *Data Category* | *Information* |  |
|  |  | Primary registry and trial identifying number | ClinicalTrials.gov, NCT04087369 | 4 |
|  |  | Date of registration in primary registry | 12 September, 2019 |  |
|  |  | Secondary identifying numbers | N/A |  |
|  |  | Source(s) of monetary or material support | This research received no specific grant from any funding agency in the public, commercial or not-for-profit sectors. The authors received no direct funding in support of this manuscript. | 23 |
|  |  | Primary sponsor | Nyaya Health Nepal |  |
|  |  | Secondary sponsor(s) | N/A |  |
|  |  | Contact for public queries | Sabitri Sapkota: sabitri.sapkota@possiblehealth.org |  |
|  |  | Contact for scientific queries | Sabitri Sapkota: sabitri.sapkota@possiblehealth.org |  |
|  |  | Public title | An integrated intervention for chronic care management in rural Nepal: a type 2 hybrid effectiveness-implementation study | 1 |
|  |  | Scientific title | An integrated intervention for chronic care management in rural Nepal: a type 2 hybrid effectiveness-implementation study | 1 |
|  |  | Countries of recruitment | Nepal | 11 |
|  |  | Health condition(s) or problem(s) studied | Type II diabetes mellitus; hypertension; chronic obstructive pulmonary disease; implementation barriers/challenges | 15; 24-26 |
|  |  | Intervention(s) | In addition to the government’s [of Nepal] planned World Health Organization Package of essential non-communicable disease interventions (WHO-PEN) program roll-out, this study will include three evidence-based components: 1) non-communicable disease (NCD) care integration using mid-level practitioners (MLPs) and community healthcare workers (CHWs); 2) clinical decision support (CDS) tools to ensure quality care in accordance with best practices; and 3) training and supervision of MLPs to provide motivational interviewing (MI) techniques for modifiable risk factor optimization, with a specific focus on tobacco and alcohol use. | Supplemental File 1 |
|  |  | Key inclusion and exclusion criteria | For primary quantitative outcomes, the study population will include adult patients (≥18 years of age) who qualify for a diagnosis of hypertension, type II diabetes, and/or COPD, according to WHO-PEN guidelines, and are engaged in longitudinal care by *Nyaya Health Nepal’*s team in Achham and Dolakha. The study will limit enrollment to the catchment areas served by both the facility-level and CHW-level services deployed by *Nyaya Health Nepal.*  Study participants… are considered engaged in longitudinal care if they have at least one follow-up hospital visit after 12 months of their initial visit.  Exclusion criteria are (1) individuals planning to migrate from the study area prior to twelve months of exposure to the intervention, or (2) individuals explicitly requesting exclusion from the study or declining to consent for the study. | 13 |
|  |  | Study type | Prospective, mixed methods, type II hybrid effectiveness-implementation study | 10 |
|  |  | Date of first enrolment | Anticipated December 2019 | 22 |
|  |  | Target sample size | 1000 | 16 |
|  |  | Recruitment status | Not yet recruiting | 22 |
|  |  | Primary outcome(s) | Condition-specific “at-goal” metrics: % of enrolled NCD patients achieving “at goal” status, at the completion of the study period  Type II diabetes mellitus: Hemoglobin A1c < 7.5 OR fasting blood sugar <130 mg/dL*  Hypertension: Blood pressure <130/80mm Hg or patient-tailored goal per risk stratification^  Chronic obstructive pulmonary disease: ≤1/3 Anthonisen criteria | 14-15; 24-26 |
|  |  | Secondary outcome(s) | Tobacco use status: % of enrolled NCD patients who were using tobacco at enrollment who are non-users or who have reduced by >50% their tobacco intake, at the completion of the study period  Alcohol use status: % of enrolled NCD patients who were alcohol drinkers at enrollment who are non-drinkers or who have reduced by >50% alcohol intake, at the completion of the study period | 15-16; 24-26 |
| Protocol version | 3 | Protocol date: 02.22.19; version 1, amendment 1, IRB protocol from Nepal Health Research Council; authors: SH, SS, DC primary reason for amendment: addition of new research team members, modification of qualitative instruments to explore risk factor modification and behaviors; addition of secondary implementation evaluation aim around exploration of risk factor modification and behaviors  Revision chronology:  Protocol date: 07.09.18, version 1, approved IRB protocol from Nepal Health Research Council; authors: DS, AK, DC, SH, DM | |  |
| Funding | 4 | This trial was conducted with no external funding and was instead funded by Nyaya Health Nepal with financial support from the Government of Nepal, Possible, and other private and philanthropic support. The authors received no direct funding in support of this manuscript. | | 23 |
| Roles and responsibilities | 5a | Dan Schwarz, MD, MPH*^1,2,3,4,5^ dan@possiblehealth.org  Santosh Dhungana, MBBS, MD-GP*^6^ santosh@possiblehealth.org  Anirudh Kumar, MD^7^ anirudh.kumar@nyulangone.org  Bibhav Acharya, MD^1,8^ bibhav.acharya@ucsf.edu  Pawan Agrawal, MBBS, MD-GP^1^ pawan.agrawal@possiblehealth.org  Anu Aryal, MPH^1,9^ anu.aryal@possiblehealth.org  Aaron Baum, PhD^10^ aaron.baum@mssm.edu  Nandini Choudhury, MPH^1,10^ nandini@possiblehealth.org  David Citrin, MPH, PhD^1,10,11,12,13^ david@possiblehealth.org  Binod Dangal, MBBS, MD-GP^1^ binod@possiblehealth.org  Meghnath Dhimal, MSc, PhD^14^ meghdhimal@gmail.com  Bikash Gauchan, MBBS, MD-GP^1^ bikash@possiblehealth.org  Tula Gupta^,^ MBBS, MD-GP^1,15^ tula@possiblehealth.org  Scott Halliday, MS^1,10,11,13^ scott@possiblehealth.org  Biraj Karmacharya, PhD, MPH, MS, MBBS^9,16,17^ birajmk@kusms.edu.np  Sandeep Kishore, MD, PhD, MSc^10,18,19^ sandeep.kishore@mssm.edu  Bhagawan Koirala, MD, FACC^20^ koiralabhagawan@gmail.com  Uday Kshatriya^1^ uday@possiblehealth.org  Erica Levine, MPH^10,18^ erica.levine@mssm.edu  Sheela Maru, MD, MPH^1,10,18,21,22^ sheela@possiblehealth.org  Pragya Rimal, MA^1^ pragya@possiblehealth.org  Sabitri Sapkota, PhD^1,10^ sabitri@possiblehealth.or  Ryan Schwarz, MD, MBA^1,2,3,22^ ryan@possiblehealth.org  Archana Shrestha, PhD^9,23,24^, archana@kusms.edu.np  Aradhana Thapa, MPH^1^, aradhana@possiblehealth.org  Duncan Maru, MD, PhD^1,10,1,8,25,26^ duncan@possiblehealth.org  *Indicates co-first authors   1. Nyaya Health Nepal, Kathmandu, Nepal 2. Division of Global Health Equity, Department of Medicine, Brigham and Women’s Hospital, Boston, MA, USA 3. Department of Medicine, Harvard Medical School, Boston, MA, USA 4. Department of Medicine, Beth Israel Deaconess Medical Center, Boston, MA, USA 5. Ariadne Labs, Harvard T.H. Chan School of Public Health and Brigham and Women’s Hospital, Boston, MA, USA 6. Department of Internal Medicine, Hurley Medical Center, Flint, MI, USA 7. Department of Medicine, NYU Langone Health, New York, NY, USA 8. Department of Psychiatry, University of California San Francisco, San Francisco, CA, USA 9. School of Medical Sciences, Kathmandu University, Dhulikhel, Nepal 10. Arnhold Institute for Global Health, Icahn School of Medicine at Mount Sinai, New York, NY, USA 11. Department of Global Health, University of Washington, Seattle, WA, USA 12. Department of Anthropology, University of Washington, Seattle, WA, USA 13. Henry M. Jackson School of International Studies, University of Washington, Seattle, WA, USA 14. Nepal Health Research Council, Ministry of Health and Population, Kathmandu, Nepal 15. Health Equity Action Leadership Initiative, University of California, San Francisco, San Francisco, CA, USA 16. Nepal Technology Innovation Center, Kathmandu University, Dhulikhel, Nepal 17. Sun Yat-sen Global Health Institute, Sun Yat-sen University, Guangzhou, China 18. Department of Health Systems Design and Global Health, Icahn School of Medicine at Mount Sinai, New York, NY, USA 19. Young Professionals Chronic Disease Network, New York, NY, USA 20. Institute of Medicine, Tribhuvan University Teaching Hospital, Kathmandu, Nepal 21. Department of Obstetrics, Gynecology, and Reproductive Sciences, Icahn School of Medicine at Mount Sinai, New York, NY, USA 22. Division of General Internal Medicine, Department of Medicine, Massachusetts General Hospital, Boston, MA, USA 23. Yale School of Public Health, Center for Methods in Implementation and Prevention Science, New Haven, CT, USA 24. Yale School of Public Health, Department of Chronic Disease Epidemiology, New Haven, CT, USA 25. Department of Internal Medicine, Icahn School of Medicine at Mount Sinai, New York, NY, USA 26. Department of Pediatrics, Icahn School of Medicine at Mount Sinai, New York, NY, USA | | 1-2; 23-24 |
|  | 5b | Nyaya Health Nepal (research performance site in Nepal implementing the programmatic intervention and carrying out the study protocol); contact: Sabitri Sapkota, Director of Implementation Research: sabitri.sapkota@possiblehealth.org | |  |
|  | 5c | N/A | |  |
|  | 5d | N/A | |  |
| Introduction |  |  | |  |
| Background and rationale | 6a | *Study aims:*  We will conduct a type 2 hybrid effectiveness-implementation trial (where effectiveness and implementation are simultaneously tested with equal priority simultaneously)[68, 69] to evaluate an integrated NCD care management intervention. The intervention will leverage the Nepali government’s planned roll-out of WHO-PEN in two rural districts. In addition to the government’s roll-out, the intervention will include three evidence-based components: 1) NCD care provision by MLPs and CHWs that is integrated between facilities and communities; 2) CDS tools for MLPs and CHWs to optimize adherence to best practices; and 3) training and supervision of MLPs in using MI to facilitate tobacco and alcohol cessation.  *Justification/rationale:*  In response to the growing need for evidence-based NCD service delivery, the World Health Organization has compiled a set of protocols within the Package of Essential Non-Communicable Disease Interventions for Primary Health Care in Low-Resource Settings (WHO-PEN). [30, 31]…  The WHO, together with the World Heart Federation, the World Stroke Organization, the United States Centers for Disease Control, the International Society of Hypertension, and the World Hypertension League, has released complementary guidelines to the WHO-PEN, called HEARTS.[32]…  Recent evidence has shown the feasibility of adopting WHO-PEN at the primary care level in LMICs, including implementation with MLPs.[33-36] These data suggest that it is feasible for to deploy WHO-PEN at the population level in primary care settings….  Clinical decision support (CDS) tools facilitate the use of algorithmic care protocols such as WHO-PEN by health workers at the point of care…  While many of the contributing etiologies for NCD epidemics are far upstream of individuals’ lives, and out of their control,[45] there are certain risk factors that are modifiable by patients, families, and health workers. Among others, it is widely documented that alcohol and tobacco consumption directly contribute to the development and progression of cardiovascular disease, diabetes, and chronic obstructive pulmonary disease (COPD).[46]…  *Background:*  The burden of non-communicable diseases (NCDs) is rising globally… there is a paucity of large-scale implementation data available to evaluate such models of care delivery, making it difficult for LMIC policy-makers to decide whether to adopt these strategies.  Description of research question and justification for undertaking the trial, including summary of relevant studies (published and unpublished) examining benefits and harms for each intervention | | 10-11; 6-8 ;5-6 |
|  | 6b | No comparators. It is not feasible nor ethically acceptable to obtain data on a comparison (control) group in this population. Given the lack of national or local NCD systems, no data are available from other sources prior to the start of the study. | | 14 |
| Objectives | 7 | The study has two Specific Aims: effectiveness (Specific Aim 1) and implementation (Specific Aim 2), as detailed in Table 1. For Specific Aim 1, the primary outcome will be the proportion of patients who meet disease-specific, evidence-based control measures at the completion of their initial twelve months engaged in treatment. These “at-goal” metrics aim to serve as simplified measures to assess disease control status, recognizing the limitations associated with multiple disease-specific metrics in settings like rural Nepal, especially for patients with multiple co-morbid conditions. These are presented in Table 2.  We hypothesize that the integrated intervention will lead to a 10% increase in the “at goal” status of the combined disease cohorts, over a twelve-month follow up period… We additionally hypothesize a 10% improvement in the status of each of the two secondary outcomes: tobacco and alcohol use, as measured by patient-reported outcomes in Table 1. | | 14-15; 18-19 |
| Trial design | 8 | Design: We will conduct a type 2 hybrid effectiveness-implementation trial (where effectiveness and implementation are simultaneously tested with equal priority simultaneously)[68, 69] to evaluate an integrated NCD care management intervention…  The study will evaluate effectiveness using a pre-post design with stepped implementation… The intervention will be implemented in a step-wise fashion in coordination with municipal-level government authorities and study staff.  Allocation: N/A. All participants/study clusters receive the intervention as there’s no control. It is not feasible nor ethically acceptable to obtain data on a comparison (control) group in this population.  Framework: We will study the implementation of the intervention utilizing both quantitative and qualitative methods applying the RE-AIM (Reach, Efficacy, Adoption, Implementation, and Maintenance) framework [79]. | | 10, 3-4, 11; 14 |
| Methods: Participants, interventions, and outcomes | | | |  |
| Study setting | 9 | The study will take place in Achham and Dolakha districts of Nepal across four municipalities…  Achham is a remote, impoverished district of 260,000 people, with large migrant populations and a history of social disruption during the Nepali civil conflict.[70-74] Achham has one of the highest district-level under-five mortality rates[75] and one of the lowest human development indices in the country.[76] The study implementers have been delivering some NCD-related care at the district-level Bayalpata Hospital and to communities within the hospital’s catchment population since 2008. Bayalpata Hospital serves approximately 90,000 outpatient and 3,000 inpatient visits per year. CHW services include proactive case detection, care coordination, and counseling. The study will include a catchment population of approximately 50,000 in Achham across two municipalities.  The second district is Dolakha, one of the hardest hit districts in the 2015 earthquakes.[77] *Nyaya Health Nepal*’s work in Dolakha is based at Charikot Primary Health Care Center, which serves approximately 60,000 outpatients per year, with similar CHW services to those in Achham’s. The study will include a population of approximately 30,000 in Dolakha across two municipalities. Thus, the total expected study population will be 80,000.  Within the context of the public private partnership between the government and *Nyaya Health Nepal*, no user fees are charged for any facility-based or community-based services, in either Achham or Dolakha, thereby mitigating financial access barriers to care delivery and study participation. | | 11-12 |
| Eligibility criteria | 10 | For primary quantitative outcomes, the study population will include adult patients (≥18 years of age) who qualify for a diagnosis of hypertension, type II diabetes, and/or COPD, according to WHO-PEN guidelines, and are engaged in longitudinal care by *Nyaya Health Nepal’*s team in Achham and Dolakha. The study will limit enrollment to the catchment areas served by both the facility-level and CHW-level services deployed by *Nyaya Health Nepal.*  Study participants… are considered engaged in longitudinal care if they have at least one follow-up hospital visit after 12 months of their initial visit.  Exclusion criteria are (1) individuals planning to migrate from the study area prior to twelve months of exposure to the intervention, or (2) individuals explicitly requesting exclusion from the study or declining to consent for the study. | | 13 |
| Interventions | 11a | In this supplemental file, we provide a detailed description of the study intervention. In addition to the government’s planned World Health Organization Package of essential non-communicable disease interventions (WHO-PEN) program roll-out, this study will include three evidence-based components: 1) non-communicable disease (NCD) care integration using mid-level practitioners (MLPs) and community healthcare workers (CHWs); 2) clinical decision support (CDS) tools to ensure quality care in accordance with best practices; and 3) training and supervision of MLPs to provide motivational interviewing (MI) techniques for modifiable risk factor optimization, with a specific focus on tobacco and alcohol use.  *Non-communicable disease care integration using MLPs and CHWs*  The NCD care management intervention focuses on integrating care delivery between the facility and community level, using MLPs at the facilities and CHWs in the communities. These settings are further linked by the EHRs and mobile phone applications, which are described in greater detail below.  In Nepal, there is already a precedent of MLPs providing the majority of outpatient care, [1] and CHWs called Female Community Health Volunteers, providing community-based service delivery,[2] but these systems have not been integrated optimally for NCD management. Our intervention strengthens the existing MLP cadre at the facility by recruiting additional staff members, providing intensive NCD-focused training on a regular basis with daily continuing medical education programs, and ensuring quality supervision by a cadre of Bachelor of Medicine, Bachelor of Surgery (MBBS) level staff physicians and senior general practitioner physicians.  The MLPs in the intervention are from the Nepali cadre of health assistants (HAs), who have three years post-secondary healthcare education. In the intervention, the primary care NCD services are delivered predominantly by HAs. HAs are responsible for algorithmic, diagnostic and treatment services driven by the WHO-PEN, in addition to health education for patients, which is described in greater detail below.  The HAs in this intervention are overseen at the facilities by a cadre of MBBS-trained staff physicians, who are, in turn, overseen by several general practitioner residency-trained physicians (called “MD-GP” physicians in the local setting). The HAs receive daily, on-going supportive supervision and continuing medical education sessions, targeted at the MLP-level of education, facilitated by the MBBS and MD-GP physicians (Supplemental File 2).  In the community, we have developed a cadre of CHWs, which has been previously described,[3, 4] The CHWs in our intervention are a novel cadre of workers in Nepal, based on global best practices and the growing body of evidence surrounding CHW program development.[5] This cadre is notably different from the pre-existing Female Community Health Volunteers, and has been developed over the past ten years as part of the public-private partnership between *Nyaya Health Nepal* and the Ministry of Health and Population. These CHWs are all female, from the communities they serve, with a minimum of tenth grade (“School Leaving Certificate”) education. They are all fully-employed and salaried, integrated into the organizational management structure. To ensure accountability and quality assurance, they are overseen by Community Health Nurses (CHNs), who are, in turn, overseen by Community Healthcare Program Associates (CHPAs). CHNs are fully-qualified nurses from the Nepali staff nurse or auxiliary nurse midwife cadres, having received either three years or eighteen months post-secondary education, respectively. All CHNs are registered with the Nepal Medical Council as licensed practitioners. The CHPAs oversee the CHNs and are, in turn, overseen by one district-level Community Healthcare District Manager. This network of CHWs has been described in greater detail previously.[3, 6, 7]  The CHWs all receive an initial healthcare-focused training at the time of hiring, and then on-going weekly continuing education and supportive supervision. The CHWs are supervised by CHNs one to two times per month, during the CHWs’ routine home-visits for patients. The system is designed to provide continual supportive supervision and improvement-focused feedback, during the course of their regular daily work responsibilities. In addition to these supervision visits, the CHWs attend at least twice-monthly meetings at central offices at their service hub, which vary in location, but are mostly based at village healthcare facilities throughout the districts. During these meetings, they have direct one-to-one review of their patient records with CHNs, and on-going training. The CHPAs oversee these meetings, providing structured teaching and mentorship to the CHNs and CHWs, reviewing the patient-related data, and identifying areas for targeted improvement.  At the community level, for NCD patients specifically, CHWs are currently trained to provide post-facility visit follow-up, reviewing medication adherence, side effect screening, and general NCD health education as provided in the PEN packages. They visit each NCD patient once monthly, and on an as-needed basis when instructed by the MLPs or requested by the patients themselves.  *Clinical decision support tools to ensure quality care in accordance with best practices*  To address continuity of care between visits, we have developed a digital network linking facility-based and community-based visits. At the facilities, MLPs utilize an EHR system that has been described previously, designed for resource-limited settings.[8] In the community, all CHWs are equipped with an Android-based mobile phone, with the CommCare application installed. By linking patient records across facility and community settings, both MLPs and CHWs have access to all patient data, decreasing the discontinuity and fragmentation associated with unlinked visits across different settings and time.  Furthermore, these digital tools enable the use of clinical decision support for MLPs and CHWs. Well-described globally, variances in quality of care[9] – the “know-do gap” – between providers and facilities, are a significant barrier to quality NCD provision at scale. Our intervention employs protocol-based algorithmic care[10-12] at both facility and community visits, designed to optimize consistent quality from visit to visit, in line with evidence-based practices. These clinical decision support tools are integrated into the EHR at facilities and within the CommCare application used by CHWs in the communities.  At the facilities, clinical decision support features guide MLPs through templated condition-specific protocols. While this intervention focuses on a limited set of specific conditions (hypertension, type II diabetes, and chronic obstructive pulmonary disease (COPD)), the EHR clinical decision support tools are designed to encompass a large set of common conditions relative to the local setting. For NCDs, these algorithms are based on PEN protocols.  In the community, CHWs use the CommCare application, an open-source mobile phone platform for healthcare workers, which has been used in over 50 countries globally, and has been studied in 39 peer-reviewed publications.[13] CommCare and other clinical decision support tools are well-equipped to provide guidance for healthcare workers providing care for which clear algorithms exist, such as the PEN protocols. CommCare includes capabilities for data collection, decision support, task reminders, and counseling tools. The mobile application can be accessed and used in the community offline and the data are uploaded at a later time when online services are accessible.  In this intervention, we have customized the CommCare application to include NCD condition-specific modules based on PEN protocols. The CHWs utilize these condition-specific templated modules during the course of their home visits, to assure that their NCD community-based services are in line with evidence-based practices. Data from the CHWs’ individual CommCare accounts are analyzed routinely by CHNs and CHPAs to provide improvement-focused feedback during their in-person meetings, as described above.  *Motivational interviewing techniques for modifiable risk factor optimization*  To provide comprehensive NCD care management, including both preventative and curative services, individual-level risk factor modification counseling is a critical aspect of the intervention. While many of our patients’ health issues are related to social and environmental factors outside the scope of healthcare alone,[14, 15] our intervention prioritizes individual-level counseling using MI techniques to improve our patients’ circumstances to prevent disease and, when present, the progression of these NCDs. This counseling is informed by WHO-PEN “brief intervention” guidance,[16] in addition to MI techniques. As a component of all facility visits, patients spend time (usually between 5-10 minutes per visit) with a dedicated NCD-specific MLP counselor. Using a standardized template, MLPs provide counseling and education about modifiable risk factors such as tobacco, alcohol, diet and nutrition; medication adherence and treatment side effects; planned follow-up needs; and other condition-specific issues. In the community, CHWs use similar protocols to discuss risk factors, medication adherence, and follow-up. | | Supp File 1, pg 1-3 |
|  | 11b | Exclusion criteria are (1) individuals planning to migrate from the study area prior to twelve months of exposure to the intervention, or (2) individuals explicitly requesting exclusion from the study or declining to consent for the study. | | 13 |
|  | 11c | The study will limit enrollment to the catchment areas served by both the facility-level and CHW-level services deployed by *Nyaya Health Nepal.* Study participants will be initially enrolled during facility-based visits at Bayalpata Hospital and Charikot Primary Health Care Center prior to the completion of intervention roll-out, and are considered engaged in longitudinal care if they have at least one follow-up hospital visit after 12 months of their initial visit. Digital health records that link between the facility-based EHR and the CHWs’ mobile-phone applications will be utilized to share patient data across settings, when available. CHWs can identify potential patients in the community and refer them to the facility for diagnosis confirmation, following which they could be included in the study. Patients’ receipt of care will not be contingent upon their enrollment in the study; all patients will continue to receive care per routine service delivery… | | 14 |
|  | 11d | Care provision will be unrelated to consent, and there will be no difference in care provision based on consent status. | | 20 |
| Outcomes | 12 | *Primary outcomes:*  Condition-specific “at-goal” metrics: % of enrolled NCD patients achieving “at goal” status, at the completion of the study period  Type II diabetes mellitus: Hemoglobin A1c < 7.5 OR fasting blood sugar <130 mg/dL*  Hypertension: Blood pressure <130/80mm Hg or patient-tailored goal per risk stratification^  Chronic obstructive pulmonary disease: ≤1/3 Anthonisen criteria  *Secondary outcomes:*  Tobacco use status: % of enrolled NCD patients who were using tobacco at enrollment who are non-users or who have reduced by >50% their tobacco intake, at the completion of the study period  Alcohol use status: % of enrolled NCD patients who were alcohol drinkers at enrollment who are non-drinkers or who have reduced by >50% alcohol intake, at the completion of the study period | | 14-16; 24-26 |
| Participant timeline | 13 | Study participants will be initially enrolled during facility-based visits at Bayalpata Hospital and Charikot Primary Health Care Center prior to the completion of intervention roll-out, and are considered engaged in longitudinal care if they have at least one follow-up hospital visit after 12 months of their initial visit. Digital health records that link between the facility-based EHR and the CHWs’ mobile-phone applications will be utilized to share patient data across settings, when available. CHWs can identify potential patients in the community and refer them to the facility for diagnosis confirmation, following which they could be included in the study. Patients’ receipt of care will not be contingent upon their enrollment in the study; all patients will continue to receive care per routine service delivery. | | 13 |
| Sample size | 14 | 1000  We calculate power based on a simplified design to compare paired proportions using a two-sided McNemar’s test with an 0.05 Type I error (alpha) level. The primary outcome is the proportion of patients who achieve their NCD control target after 12 months of being engaged in care. Based on previous analyses and assuming an attrition rate of 30%, we anticipate that a total of at least 1,000 adult patients across both sites will be available to analyze in the study. | | 16 |
| Recruitment | 15 | For primary quantitative outcomes, the study population will include adult patients (≥18 years of age) who qualify for a diagnosis of hypertension, type II diabetes, and/or COPD, according to WHO-PEN guidelines, and are engaged in longitudinal care by *Nyaya Health Nepal’*s team in Achham and Dolakha. The study will limit enrollment to the catchment areas served by both the facility-level and CHW-level services deployed by *Nyaya Health Nepal.* Study participants will be initially enrolled during facility-based visits at Bayalpata Hospital and Charikot Primary Health Care Center prior to the completion of intervention roll-out, and are considered engaged in longitudinal care if they have at least one follow-up hospital visit after 12 months of their initial visit. Digital health records that link between the facility-based EHR and the CHWs’ mobile-phone applications will be utilized to share patient data across settings, when available. CHWs can identify potential patients in the community and refer them to the facility for diagnosis confirmation, following which they could be included in the study. Patients’ receipt of care will not be contingent upon their enrollment in the study; all patients will continue to receive care per routine service delivery. This represents an exhaustive convenience sampling method as all eligible patients identified at Bayalpata Hospital and Charikot Primary Health Care Center may be enrolled in the study. | | 13 |
| **Methods: Assignment of interventions (for controlled trials)** | | | |  |
| Allocation: |  |  | |  |
| Sequence generation | 16a | N/A | |  |
| Allocation concealment mechanism | 16b | N/A | |  |
| Implementation | 16c | N/A | |  |
| Blinding (masking) | 17a | N/A | |  |
|  | 17b | N/A | |  |
| **Methods: Data collection, management, and analysis** | | | |  |
| Data collection methods | 18a | Quantitative data for patient outcomes will be extracted from the facility-based EHR and the CHW’s mobile phone application (Supplemental File 1), and will be used to assess Specific Aims 1 and 2. (Table 1) All implementation-related data for evaluating the performance of MLPs and CHWs (Table 1) will be collected by the responsible MLP and CHW supervisors in digitized checklists within the EHR and mobile phone application…  Costing data for the intervention will be collected utilizing a “top-down” method, as described by the Joint Learning Network[83]. This method will document direct and indirect costs associated with the NCD care delivery intervention described here and related administrative functions (including planning and administration; training; supervision and monitoring and evaluation; data management; and continuous surveillance) will be disaggregated. For the purposes of this pragmatic study, this methodology will be appropriate to estimate the additional marginal costs of the intervention (rather than cost-savings or secondary cost implications) as compared to general standard of care…  Qualitative data will be used for Specific Aim 2. (Table 2) Staff members, patients, community leaders, and government officials will be approached for KIIs and FGDs. Purposive sampling will be used, aiming to maximize heterogeneity across sex, socioeconomic position, healthcare issues, geographic location, age, caste-class, and other attributes.  KII and FGD guides will be developed in advance, and will vary across the study period, exploring specific topics of concern. A locally validated, seven-domain framework of healthcare delivery analysis will be used to inform data collection.[84] These seven domains include health service operations, supply chains, equipment, personnel, outreach, societal factors, and structural factors. Qualitative data collection will focus on these areas to assess the implementation of the intervention.  FGDs and KIIs will occur prior to the initiation of the intervention, and in intervals of six months throughout the study period, to assess on-going implementation status. All sessions will be conducted in Nepali.  [See Supplemental File 4 for preliminary data collection tools and qualitative instruments] | | 17-18, Supp File 4 |
|  | 18b | The study will limit enrollment to the catchment areas served by both the facility-level and CHW-level services deployed by *Nyaya Health Nepal.* Study participants will be initially enrolled during facility-based visits at Bayalpata Hospital and Charikot Primary Health Care Center prior to the completion of intervention roll-out, and are considered engaged in longitudinal care if they have at least one follow-up hospital visit after 12 months of their initial visit. Digital health records that link between the facility-based EHR and the CHWs’ mobile-phone applications will be utilized to share patient data across settings, when available. CHWs can identify potential patients in the community and refer them to the facility for diagnosis confirmation, following which they could be included in the study. Patients’ receipt of care will not be contingent upon their enrollment in the study; all patients will continue to receive care per routine service delivery. | | 13 |
| Data management | 19 | Quantitative data for patient outcomes will be extracted from the facility-based EHR and the CHW’s mobile phone application (Supplemental File 1), and will be used to assess Specific Aims 1 and 2. (Table 1) All implementation-related data for evaluating the performance of MLPs and CHWs (Table 1) will be collected by the responsible MLP and CHW supervisors in digitized checklists within the EHR and mobile phone application. Access to protected health information will be controlled and defined by user access groups according to clinician status. Data to be analyzed will be extracted via secure data queries from the EHR system in aggregate, partially de-identified form with external researchers signing a data sharing and use agreement prior to analysis. Cleaned, de-identified datasets will be made publicly available via a data repository….  All qualitative data will be stored on a Research Electronic Data Capture (REDCap) database.[85] REDCap user access will be defined so that researchers only have access to de-identified study data. Any paper copies of data forms will be stored in locked cabinets inside locked rooms at district facilities. Once all data are fully transcribed and validated for quality, all paper copies will be destroyed. REDCap data will be deleted twelve months after the completion of the study period…  Data from KIIs and FGDs will transcribed and coded using Grounded Theory Methodology.[86, 87] NVivo software will be used for qualitative data analysis.[88] | | 17-18, 20 |
| Statistical methods | 20a | We will use conditional multivariable logistic regression to assess patient outcomes at 12 months follow up, adjusting for potential confounding by patients’ demographics (including age, gender, caste), municipality, district, mean distance to the hospital, and engagement in care (defined as number of facility-based and community-based encounters). We additionally hypothesize a 10% improvement in the status of each of the two secondary outcomes: tobacco and alcohol use, as measured by patient-reported outcomes in Table 1.  As a secondary analysis for Specific Aim 1, namely the time-varying nature of the outcomes, we will assess the effect of the intervention, as measured in three-monthly intervals, throughout the study period, compared to baseline statistics at the time of each village-cluster enrollment. Variables will be considered as both nominal and continuous (linear effect) predictors, and the generalized linear model framework will be used to estimate effect of time-varying repeated measure intervention implementation over the several steps of the wedged design. Differential impact from time of intervention will be evaluated with test of month × intervention interaction. Models will be fit using generalized estimating equations, e.g., using SAS Proc Genmod, to calculate valid standard errors in the presence of repeated measures over time and possibly correlated outcomes at the municipality level. Assumptions of over- or under-dispersion will be examined closely, and an estimated scale parameter or negative binomial models will be used as needed.  In this mixed-methods study, Specific Aim 2 will be assessed using the RE-AIM framework for implementation trials.[79] A full list of metrics, separated by each domain of the RE-AIM framework, is presented in Table 1. Additional details regarding the supervision and audit structure for MLPs and CHWs can be found in Supplemental File 1 and 2.  For the (M)aintenance of the intervention, we will assess the costs of the intervention, using the Joint Learning Network methodology[83]. Cost data will be analyzed and presented (Table 1) to help program planners and policy makers understand the implications for possible scale of a similar intervention by the government or other entity in the future.  For quantitative data within Specific Aim 2, a similar methodology of generalized estimating equations, as described above in the section on Specific Aim 1 analysis, will be applied. Data will be assessed in three-month intervals.  For qualitative data within Specific Aim 2, analysis will be on-going and iterative, so as to continually inform further qualitative data collection, focusing on timely and relevant implementation issues. Data from KIIs and FGDs will transcribed and coded using Grounded Theory Methodology.[86, 87] NVivo software will be used for qualitative data analysis.[88] | | 19-20 |
|  | 20b | N/A | |  |
|  | 20c | N/A | |  |
| **Methods: Monitoring** | | | |  |
| Data monitoring | 21a | N/A  The intervention will leverage the Nepali government’s planned roll-out of WHO-PEN in two rural districts. In addition to the government’s roll-out, the intervention will include three evidence-based components: 1) NCD care provision by MLPs and CHWs that is integrated between facilities and communities; 2) CDS tools for MLPs and CHWs to optimize adherence to best practices; and 3) training and supervision of MLPs in using MI to facilitate tobacco and alcohol cessation. | | 10-11 |
|  | 21b | N/A | |  |
| Harms | 22 | There are minimal risks posed to patients, staff, or other key informants. The predominant risk is disclosure of protected health information, and/or qualitative data from KIIs or FGDs. All patient information will be stored on secure databases, and data access privileges will be heavily restricted. Unless otherwise deemed necessary for a specific analysis, all analyses will be conducted using a limited dataset. Qualitative data will be stored and protected as described above. | | 21 |
| Auditing | 23 | N/A | |  |
| Ethics and dissemination | | | |  |
| Research ethics approval | 24 | This study has been approved by the Ethical Review Board of the Nepal Health Research Council (#177/2018). | | 20 |
| Protocol amendments | 25 | Protocol modifications will be promptly communicated to the IRB and on the trial registry website by members of the research study team. | | 20-21 |
| Consent or assent | 26a | Within the study, all patients will provide verbal informed consent to have their de-identified data analyzed and published. Care provision will be unrelated to consent, and there will be no difference in care provision based on consent status. Verbal informed consent will also be provided by all KII and FGD participants. No incentives will be provided to study participants, to avoid any conflict of interest or coercion to participate. | | 20 |
|  | 26b | N/A | |  |
| Confidentiality | 27 | Quantitative data for patient outcomes will be extracted from the facility-based EHR and the CHW’s mobile phone application (Supplemental File 1), and will be used to assess Specific Aims 1 and 2. (Table 1) All implementation-related data for evaluating the performance of MLPs and CHWs (Table 1) will be collected by the responsible MLP and CHW supervisors in digitized checklists within the EHR and mobile phone application. Access to protected health information will be controlled and defined by user access groups according to clinician status. Data to be analyzed will be extracted via secure data queries from the EHR system in aggregate, partially de-identified form with external researchers signing a data sharing and use agreement prior to analysis…  All qualitative data will be stored on a Research Electronic Data Capture (REDCap) database.[85] REDCap user access will be defined so that researchers only have access to de-identified study data. Any paper copies of data forms will be stored in locked cabinets inside locked rooms at district facilities. Once all data are fully transcribed and validated for quality, all paper copies will be destroyed. REDCap data will be deleted twelve months after the completion of the study period. | | 17-18 |
| Declaration of interests | 28 | PA, AA, DC, BD, BG, TG, UK, PR, SS, and AT are employed by and DS, BA, NC, SH, SM, RS, and DM work in partnership with a nonprofit healthcare company (*Nyaya Health Nepal,* with support from the US-based nonprofit, *Possible*) that delivers free healthcare in rural Nepal using funds from the Government of Nepal and other public, philanthropic, and private foundation sources. DS and RS are employed at an academic medical center (Brigham and Women’s Hospital) that receives public sector research funding, as well as revenue through private sector fee-for-service medical transactions and private foundation grants. DS and RS are faculty members at a private medical school (Harvard Medical School). DS is employed at an academic medical center (Beth Israel Deaconess Medical Center) that receives public sector research funding, as well as revenue through private sector fee-for-service medical transactions and private foundation grants. DS is employed at an academic research center (Ariadne Labs) that is jointly supported by an academic medical center (Brigham and Women’s Hospital) and a private university (Harvard T.H. Chan School of Public Health) via public sector research funding and private philanthropy. SD is a medical resident at a private academic medical center (Hurley Medical Center) that receives revenue through private sector fee-for-service medical transactions and a charitable private foundation. AK is a medical resident at a private academic medical center (NYU Langone Health) that receives public sector research funding, as well as revenue through private sector fee-for-service medical transactions and private foundation grants. BA is a faculty member at a public university (University of California, San Francisco). AA is a fellow supported by a public-sector research fellowship affiliated at and BKarmacharya and AS are faculty members at a private university (Kathmandu University). DC is a faculty member, SH is a graduate student at, and DC and SH are employed part-time at a public university (University of Washington). AB, DC, SK, SM, SS, and DM are faculty members at, and NC SH, and EL are employed by a private medical school (Icahn School of Medicine at Mount Sinai). MD is employed by the Government of Nepal (Ministry of Health and Population, Nepal Health Research Council). TG is a fellow with a bidirectional fellowship program (HEAL Initiative) that is affiliated with a public university (University of California, San Francisco) that receives funding from public, philanthropic, and private foundation sources. BKarmacharya is a faculty member at a public research university (Sun Yat-sen University). SK is the founding Executive Director at an advocacy and leadership network (Young Professionals Chronic Disease Network) that receives funding from individual philanthropy. SK serves as a consultant for Resolve To Save Lives on hypertension treatment and leads a partnership on multiple chronic conditions through his institution and Teva Pharmaceuticals. BKoirala is a faculty member at a public university (Tribhuvan University, Institute of Medicine). SM is a voting member on the Board of Directors with Group Care Global, a position for which she receives no compensation. RS is employed at an academic medical center (Massachusetts General Hospital) that receives public sector research funding, as well as revenue through private sector fee-for-service medical transactions and private foundation grants. AS is a faculty member at a private university (Yale School of Public Health). DM is a non-voting member on *Possible*’s Board of Directors, a position for which he receives no compensation. All authors have read and understood Trial’s policy on declaration of interests, and declare that we have no competing financial interests. The authors do, however, believe strongly that healthcare is a public good, not a private commodity. | | 22-23 |
| Access to data | 29 | The datasets supporting the conclusions of the study will be made publicly available in de-identified form upon conclusion of the study. The final trial dataset (in limited identifier format) will be accessible to researchers at the research performance site in Nepal—Nyaya Health Nepal—and co-investigators assisting with data analysis. Participating research institutions will enter into data sharing agreements (namely between the research performance site in Nepal—Nyaya Health Nepal—and any foreign institutions where investigators are assisting with data analysis) covering terms of access to specific limited datasets; provisions for storing, sharing, and using data; and methods for securing data transfer. | | 22-23 |
| Ancillary and post-trial care | 30 | N/A | |  |
| Dissemination policy | 31a | Domestically within Nepal, six-monthly update meetings will be held between researchers and the Ministry of Health and Population to review on-going results. When completed, results of the study will be presented at the annual National Summit of Health and Population Scientists, organized by the Nepal Health Research Council, and at other relevant international conferences. Peer-reviewed publications will be drafted for international dissemination. | | 21 |
|  | 31b | N/A | |  |
|  | 31c | The datasets supporting the conclusions of the study will be made publicly available in de-identified form upon conclusion of the study. | | 22 |
| Appendices |  |  | |  |
| Informed consent materials | 32 | [See supplemental file 3 for English and Nepali version] | | Supp File 3 |
| Biological specimens | 33 | N/A | |  |

*It is strongly recommended that this checklist be read in conjunction with the SPIRIT 2013 Explanation & Elaboration for important clarification on the items. Amendments to the protocol should be tracked and dated. The SPIRIT checklist is copyrighted by the SPIRIT Group under the Creative Commons “[Attribution-NonCommercial-NoDerivs 3.0 Unported](http://www.creativecommons.org/licenses/by-nc-nd/3.0/)” license.
