# Supplementary material for: An integrated intervention for chronic care management in rural Nepal: protocol of a type 2 hybrid effectiveness-implementation study
Source: Trials. 2020 Jan 29;21:119. doi: 10.1186/s13063-020-4063-3 (PMC6990567; doi:10.1186/s13063-020-4063-3)
Supplement: Supplementary file 5 — Additional file 5. Interview guides and data collection tools. [file 13063_2020_4063_MOESM5_ESM.pdf]

PEN-PLUS: Implementation of Modified WHO PEN by Mid-Level Providers and Community Healthcare Workers in Achham and Dolakha

Data Collection Tools

1. **Implementation Checklist**

Implementation	***NPHW***
**Clinical Practice**	
Proportion trained in Protocol 1 (HTN/DM)	CHW/MLP
Proportion showing competence in Protocol 1 (HTN/DM)	CHW/MLP
Proportion of EHR templates with completed PEN/MLP CDS for HTN/DM	MLP
Proportion of CommCare extracts with completed PEN CDS in Protocol 1	CHW
Proportion trained in Protocol 3.1 (COPD)	CHW/MLP
Proportion showing competence in Protocol 3.1 (COPD)	CHW/MLP
Proportion of EHR templates with completed PEN/MLP CDS for COPD	MLP
Proportion of CommCare extracts with completed PEN CDS in Protocol 3.1	CHW
Proportion trained in Protocol 3.2 (Asthma)	CHW/MLP
Proportion showing competence in Protocol 3.2 (Asthma)	CHW/MLP
Proportion of EHR templates with completed PEN/MLP CDS for Asthma	MLP
Proportion of CommCare extracts with completed PEN CDS in Protocol 3.2	CHW
**Counseling**	
Proportion trained in Protocol 2 (Health Education)	CDC/CHW
Proportion attended four MI training sessions	CDC/CHW
Proportion filled out MI section of CD template	CDC
Proportion of CD encounters where CHW employs MI (per CHN)	CHW
**Technology**	
Average Views: Number of aggregate dashboard views / number of CHWs	CHW
Average Views: Number of aggregate dashboard views / number of MLPs	MLP
Number of performance reviews using dashboard for feedback	CHW/MLP

2. Qualitative Interview/FGD Guides

Acceptability/Feasibility Trial
Government Stakeholders Guide

1. **How do you feel about the scalability of this intervention?**
2. **What is the scope for non-physician healthcare workers to learn motivational interviewing successfully?**

Non-physician Healthcare Workers Guide

1. **(CHWs) How does active screening for hypertension compare to your pre-existing tasks?**
2. **Is there anything that makes learning these new protocols difficult?**
3. **Is there anything that makes learning them easy?**
4. **How do you feel about using the clinical decision support tool?**
    a. How has it been useful?
    b. How has it hindered your work?

<u>Patient Guide</u>

1. **Have the clinicians behaved any differently because of the clinical decision support tool?**
2. **How does the CHW's skill level compare to before?**
   a. Is the care they are providing different now than before?

Effectiveness/Implementation Trial

<u>Patient Interview Guide</u>

1. **How has your health been recently? Please describe.**
   a. Has it improved or gotten worse recently? Why?
   b. Any events that stand out?

2. **How is your disease affecting you and your family?**
   a. Do you receive support from your family? How?
   b. Has this caused sacrifice for other family members? How?
   c. How has your work or school or those of your family, been affected?
   d. What does the future look like for you and your family considering your illness?
   e. What worries you and your family most?

3. **How do you feel about the treatment and other activities you must do to manage your illness?**
   a. Why has it been hard to follow your treatment?
   b. Why has it been easy to follow your treatment?
   c. Please describe your experience with taking your medications?
   d. How do you feel about the lifestyle (diet, exercise, etc.) changes that were part of your treatment?
   e. What has been most helpful to you in maintaining your health? Least helpful?

4. **How do you feel about the way your health is being managed by the healthcare workers?**
   a. Do you feel confused, treated poorly or too quickly, unsure of what you need to do next? Do you often have many questions afterwards that are unanswered?
   b. How well do you feel treated by the CHWs?
   c. How well do you feel treated by the doctors, nurses, health assistants at the Hospital and/or Primary Health Center?
   d. Please describe your experience with attending follow-up appointments?
   e. What has been going well?
   f. What has been difficult?

5. **How do you feel those in your community help or hinder you in your illness?**
   a. What do you think others in your community think about illnesses like yours?
   b. How have you been supported by others in your community?
   c. Does this create challenges for you?

6. **What would you like to see changed that would help you most with this disease?**
   a. In the community?
   b. Within your family?
   c. At the clinics or hospital?
   d. From doctors, nurses, CHWs or other health care providers?

CHW Focus Group Discussion Guide

1. **How do you think chronic diseases are perceived in your community?**
    a. Does everyone see them the same? How?
    b. How do they affect different people more or less?
    c. How do chronic diseases have an impact on your community?
    d. Are people supportive of each other?
    e. How do you think these perspectives affect those who are sick?
    f. How does this influence your motivation in your work?

2. **What challenges do community members face in successfully managing their disease?**
    a. Which challenges create the largest impact? Which are most common?
    b. Who has most difficulty dealing with chronic disease? Who has least difficulty?
        i. How are challenges different for different people?
    c. Are those who are sick well supported by their families? By other members in the community?
    d. Which challenges do people have with their treatment program?
        i. Medications
        ii. Changes in their behaviors, such as salt, fat, sugar reduction
        iii. Attending follow-up appointments
    e. Which challenges do people have in interacting with:
        i. health providers – such as doctors, nurses, health assistants, CHWs?
        ii. The health system and its organization?

3. **What challenges do/will you face as a CHW responsible for chronic disease care?**
    a. Regarding patients or their families?
    b. Do you have enough time or resources for this work? Please explain.
    c. Do you feel you have sufficient training and knowledge? Please explain.
    d. How do you or the CHWL prioritize chronic disease work in your overall workload?
    e. What discourages you?
    f. What motivates you?

4. **How do you feel about the way chronic disease management is organized by Nyaya Health?**
    a. Do you feel well supported and prepared?
    b. Does the way care is organized create challenges for you in your work?
    c. Are challenges created for patients because of how care is organized?
    d. How do you feel about the coordination and collaboration between CHWs and other staff? Between the CHWs, primary health centers, and hospital?

5. **What other community resources could be used to improve chronic disease care and prevention?**
    a. Individuals – including past patients?
    b. Any groups from the community?

6. **What would you like to see change that would make an impact on chronic disease care for those in your community?**
    a. To make your job easier?
    b. That would most benefit patients and their families?

Staff Member Interview Guide

1. **What place do you see for chronic disease care in Nyaya Health?**
    a. Is there support or motivation for improving this care in the organization?
    b. What prioritization does this care hold?

2. **What challenges and strengths do you see at the community level for successful chronic disease management?**
    a. For patients?
    b. For CHWs?
    c. Regarding community opinion or perspectives?
    d. Social, structural, logistical, or environmental issues?
    e. What further community resources could be leveraged to support this care?

3. **What organizational challenges and strengths do you see as important to successful chronic disease management?**
    a. In terms of management?
    b. In terms of supervision?
    c. In linking and coordinating between the hospital and community health department?
    d. In shifting responsibility to CHWs, health assistants, health posts?

4. **What major issues do you see in the current system of care?**
    a. How can these be addressed?

5. **What would you like to see changed that would have an impact on improving chronic disease care and prevention?**
    a. How feasible do you think such changes would be?
    b. Which improvements should take priority and why?

6. **Qualitative Efficacy Evaluation (probes)**
    Clinical Practice
    a. Barriers/facilitators of clinical algorithms + skills implementation (all NPHWs)
    b. Usability/feasibility of CDS (all NPHWs)
    c. Provider perspectives on CDS efficacy
    Counseling
    d. Provider perspectives on challenges/opportunities of MI
    e. Patient perspectives on challenges/opportunities of MI
    Technology
    f. Usability/feasibility of performance dashboard (CHNs and physicians/MLP trainers)
    g. NPHW manager perspectives on efficacy of performance dashboard (CHNs and physicians/MLP trainers)
    h. NPHW perspectives on efficacy of performance dashboard (all NPHWs)

बिरामी अन्तरवाताा निर्देशिका

1. **हालै तपाईको स्वास््य कस्तो रहेको छ? कृपया बिस्तृत रुपमा भन्िुहोला |**

a. हालै तपाईको स्वास््य सुधार भएको छ कक खराब भएको छ? ककन?

b. यस्तो गराउने कुनै टनाहरु सम्झन लायकका छन्?

2. **तपाईको रोगले तपाई र तपाईको पररवारलाई कसरी असर गरेको छ?**

a. अदहले र पदहले िुवै बेला

b. के तपाईलाई आफ्नो पररवारको सहयोग र समथघन प्राप्त छ? कसरी? ककन छ वा ककन छैन?

c. के तपाईको रोगको कारणले तपाईको पररवारमा कसैले कुनै त्याग गनुघ परेको छ? कसरी?

d. यस रोगको कारणले कसरी तपाइको वा तपाइको पररवारको काम वा स्कूलमा असर परेको छ?

e. यस रोगलाई ववचारमा राख्िा तपाई वा तपाईको पररवारको भववष्य कस्तो िेखखन्छ?

f. तपाई वा तपाईको पररवारलाई के कुराले सबैभन्िा बढी अत्याउँछ?

3. **तपाईलाई आफ्िोरोगको उपचार र व्यवस्थापि गिाको लागग गिा भनिएका क्रियाकलापको िारेमा के धारणा छ?**

a. तपाईलाई आफ्नो उपचारका कियाकलाप गनघ ककन अप््यारो छ?

b. तपाईलाई आफ्नो उपचारका कियाकलाप गनघ ककन सजिलो छ?

c. कृपया औषधीहरु शलंिाका तपाईका अनुभवहरु सुनाउनुहोस् |

d. रोग व्यवस्थापनको लागग गनघ भननएका िीवनशैली पररवतघन (खाना, व्यायाम, आदि) को बारेमा तपाईको के धारणा छ?

e. रोग व्यवस्थापनको लागग सबैभन्िा सजिलो परेको कुरा के हो? अनन सबैभन्िा गाह्रो?

4. **तपाईको रोग व्यवस्थापिको लागग स्वास््यकमीहरुले अपिाएको तररका/ववगधको िारेमा तपाईको के धारणा छ?**

a. के तपाई आफ्नो उपचारको बारेमा झुजककनु भएको छ, वा अब के गने होला भनेर ननजचचत नहुनु भएको छ? के तपाईलाई आफ्नो उपचार धेरै चाँडो भएको वा कम गुणस्तरीय भएको िस्तो लाग्छ ? के स्वस्थायाकशमघलाई भेदटसके पनछ पनन धेरै प्रचनहरु अनुत्तरत रहने गरेका छन्?

b. सामुिानयक स्वास््य ववभाग र मदहला सामुिानयक स्वास््य स्वयंसेवीकाबाट (FCHVs) प्राप्त परामिघको बारेमा तपाईको के धारणा छ?

c. सामुिानयक स्वास््य कायघकताघ नेतृ (CHWL) / मदहला सामुिानयक स्वास््य स्वयंसेवीकाले तपाईलाई कनतको राम्रो व्यवहार गरे िस्तो लाग्छ ?

d. अस्पताल र/वा सब/हेल्थ पोस्टमा डाकटर, हेल्थ अशसस्टेन्ट, नसघ, आदिले तपाईलाई कनतको राम्रो व्यवहार गरे िस्तो लाग्छ ?

e. फलोअपको लागग आउँिाको तपाईको अनुभव कस्तो रह्यो ?

f. के कुरा राम्रो भइरहेको छ?
g. के कुरा गाह्रो भइरहेको छ?

5. सामुर्दानयक स्वास््य कायाकतााले तपाईको स्वास््यमा पारेको असरको िारेमा तपाईको के धारणा छ?
a. सबैभन्िा सहयोगीमुलक के भयो?
b. सबैभन्िा सहयोगीमुलक के भएन?
c. सामुिानयक स्वास््य ववभाग र मदहला सामुिानयक स्वास््य स्वयंसेवीकाबाट (FCHVs) प्राप्त परामिघको बारेमा तपाईको के धारणा छ?
d. सामुिानयक स्वास््य कायघकताघ नेत्रृ (CHWL) / मदहला सामुिानयक स्वास््य स्वयंसेवीकाले तपाईलाई कनतको राम्रो व्यवहार गरे िस्तो लाग्छ ?
e. ननम्न कुराहरुमा वहाँहरुले कसरी असर पानुघ भएको छ?
i. तपाईले अस्पतालमा आएर गनुघपने फलोअपमा
ii. आफ्नो रोगको बारेमा तपाईको बुझाईमा
iii. तपाईले शलने औषधी र त्यसको प्रयोगको बारेमा
iv. तपाईको िीवनिैलीमा ताकी तपाई स्वस्थ रहन सकनुहोस् र अझ बबरामी नहुनुहोस् भन्ने बारेमा

6. **तपाइको धारणामा तपाईको समुर्दायका सर्दस्यहरुले तपाईको रोग व्यवस्थापिमा कसरी सहयोग वा अवरोध पुयााउि भएको छ?**
a. तपाईको िस्तो रोगको बारेमा समुिायका अरु सिस्यहरुको के धारणा छ?
b. समुिायबाट तपाईलाई कसरी सहयोग भएको छ?
c. के यसले गिाघ तपाईलाई केदह समस्या परेको छ?

7. **तपाईलाई आफ्िो रोग व्यवस्थापि गिा कस्तो खालको पररवताि भए सिैभन्दा सजिलो हुन््यो िस्तो लाग्छ?**
a. समुिायमा?
b. तपाईको पररवार शभत्र?
c. हेल्थ पोस्ट वा अस्पतालमा?
d. डाकटर, नसघ, सामुिानयक स्वास््य कायघकताघ तथा अन्य स्वास््यकममीहहरुबाट

सामुिानयक स्वास््य कायघकताघ -नेत्रृ (*CHW*) गहन सामुदहक छलफल (Focus Group Discussion) ननिेशिका

कायघिम-पूवघ ननिेशिका

1. **तपाईको समुर्दायमा दर्दर््ा रोगलाई कसरी हेररन्छ ?**
a. के माननसहरुले यसको बारेमा सामान्यतया वा महत्वपूणघ छ भनेर हेछघन्?

i. दिर्घ र तीब्र रोगको बारेमा माननसहरुले कसरी एउटै वा शभन्न तररकाले सोच्छन्?
ii. दिर्घ रोग्ल्को उपचारमा परम्परागत वा बैकजल्पक उपचार प्रणालीको के भूशमका रहेको छ? के दिर्घ रोगकोलागग के यो उपचार प्रणाली कम वा बढी सान्धववघक छ?
b. के सबैिनाले यो रोगलाई एकै रुपमा हेछघन? कसरी?
c. दिर्घकाशलन रोगले तपाईको समुिायमा केदह प्रभाव पारेको छ?
d. दिर्घकाशलन रोगले तपाईको समुिायमा कसरी प्रभाव पारेको छ?
e. के बबरामीहरुलाई नतनीहरुको पररवारको राम्रो सहयोग रहन्छ? समुिायका अन्य माननसहरुबाट? के पररवार/समुिायका सबै रोगीलाई बराबर प्रभाव पिघछ?
f. पररवारका सिस्य र समुिायका माननसले हेने दृस्टीकोणले बबरामीलाई कस्तो प्रभाव/असर पिघछ?
g. यसले तपाईको काम गने उत्प्रेरणालाई कसरी प्रभाव पािघछ?

2. **समुर्दायका मानिसहरुलाई आफ्िो दर्दर्ा रोगको सफल व्यवस्थापिका चुिौनतहरु के के छ्ि?**
a. ती मध्ये कुन चादहँ सबैभन्िा बढी प्रभाव पािघछ? कुन चादहँ सबैभन्िा सामान्य/सधैं सामना गरररहनु पने हो?
b. कसलाई सबैभन्िा बढी र कसलाई सबैभन्िा कम प्रभाव पािघछ? कसरी? ककन?
i. मदहला, पुरुष, बालबाशलका, बृद्ध, मानशसक रोगग?
c. आफ्नो उपचारको िममा उनीहरुलाई कस्ता चुनौनतहरु सामना गनुघपछघ?
i. ननयशमत औषधी सेवन गनघ
1. बबरामीको औषगध सेवन गने बनन कसरी सुधानघ सककन्छ?
2. के पररवारले बबरामीलाई सम्झाउने वा िबाब दिने गछघ?
ii. बानीहरु सुधार गने, िस्तै – नुन, गचल्लो, गुशलयो कम खाने, चुरोट कम वपउने
iii. फलोअपको लागग ननयशमत आउने
d. बबरामीहरुलाई ननम्नहरुसिंग अन्तरकिया गनघ के चुनौतीहरु छन्:
i. स्वास््यकममीह – िस्तै डाकटर, नसघ, हेल्थ अशसस्टेन्ट, मदहला सामुिानयक स्वास््य स्वयिंसेववका ?
ii. स्वास््य सिंस्था र यसको व्यवस्थापनसिंग?

3. **अदहले न्याय हेल्थ र सरकारले गरररहेको दर्दर्ा रोग व्यवस्थापिको तररकाको िरेमा तपाईको के धारणा छ?**
a. के यसलाई पूणघ सहयोग र तयारी छ?
b. के दिर्घ रोग व्यवस्थापन महत्वपूणघ छ? ककन छ वा छैन?
c. बबरामीको उपचारको सिंरचनाले गिाघ के बबरामीहरुलाई अप््यारो पारेको छ?
d. के उपचारको सिंरचनाले गिाघ तपाईहरुको काममा पनन चुनौनतहरु शसिघना भएका छन्?
e. म.सा.स्वा.स्व. र अन्य कमघचारी बबचको समन्वय र सहकायघको बारेमा के ववचार छ?
म.सा.स्वा.स्व./सा.स्वा.का.ने, हेल्थ पोस्ट र अस्पतालको बबचको?
f. हेल्थपोस्टका कमघचारीसिंग तपाइको कस्तो सम्बन्ध छ?
g. के तपाइलाई कदहलेकाहीँ न्याय हेल्थले अहाघएको काम गाह्रो वा असिंधववघक लाग्छ? ककन? यस कुरालाई तपाईले कसरी सामना गनुघ हुन्छ?

4. अन्तवाातााकारको लागग

दिर्घ रोग व्यवस्थापनको लागग योिना गरराएका पररवतघनको बारेमा बताउनुहोस् |

 जकलननकल फलोअप र परामिघको बारेमा अझै बढी मानक *(standard)* प्रोटोकलहरु लागु गने
 सामुिानयक स्वास््य ववभागबाट परामिघलाई वविेष िोड दिने –स्वास््यकमिमीहले दिर्घ रोगका बबरामीहरुलाई सा.स्वा.बब. पठाउनु पने
 हेल्थ पोस्टका कमघचारीलाई सानानतना दिर्घ रोगलाई अनुगमन, खोि, फलोअप, तथा प्रेषण गनघको लागग ताशलमको व्यवस्था गने
 दिर्घ रोगका बबरामीलाई फलोअपको लागग आउन सम्झाउन, स्वास््य परामिघलाई सुद्रीढ बनाउन, औषधी सेबनको बारेमा िान्न, तथा नयाँ बबरामीहरुको पदहचान गनघ म.सा.स्वा.स्व.को पररचालन गने
 बबरामीको आफ्नो हेरचाह र रोग व्यवस्थापन आफैं गनघ िोड दिने

5. **न्याय हेल्थले दर्दर्् रोग व्यवस्थापिको संरचिामा ल्याउि लागेको पररवतािको िारेमा तपाईको के धारणा छ?**
a. के तपाईलाई दिर्घ रोगको फलोअप तपाईको िागगरको अंिि हुनुपछघ िस्तो लाग्छ? ककन लाग्छ वा ककन लाग्िैन?
b. के म.सा.स्वा.स्व./सा.स्वा.का.नेका यी भूशमकाहरु तपाईलाई उपयुकत छन्? ककन छ वा ककन छैन?
c. यी कायघहरु गनघ तपाईलाई केके कुराको आवचयकता पछघ?
d. दिर्घ रोगका बबरामीको सहयोगको लागग म.सा.स्वा.स्व./सा.स्वा.का.ने ले खेल्ने भूशमकाको बारेमा के कुरा बविेष छ िस्तो लाग्छ?
e. बबरामीहरुले म.सा.स्वा.स्व./सा.स्वा.का.ने को भ्रमणबाट के फाइिा हुन्छ िस्तो लाग्छ?
f. के बबरामीहरु आफ्नो आफ्नो हेरचाह र रोग व्यवस्थापन आफैं गनघ सकछन िस्तो लाग्छ? ककन सकछन वा ककन सकैनन्?
i. औषगध ननयशमत सेवन गने, आफ्नो बाननहरुको सुधार गने, आफ्नो रोगको बारेमा िान्न खोज्ने, र फलोअपमा ननयशमत रुपमा आउने?

6. दर्दर्् रोग फलोअप र र्-भ्रमणको लागग जिम्मेवार म.सा.स्वा.स्व./सा.स्वा.का.िेको भूशमकामा तपाई के चुिौती र्देख्िु हुन्छ?
a. तपाईको काममा के कुरा सबैभन्िा सजिलो छ वा हुन्छ?
b. तपाईको काममा के कुरा सबैभन्िा गाह्रो छ वा हुन्छ?
c. बबरामी र वहाँहरुको पररवारबाट के चुनौतीहरु सामना गनुघपनघ सकछ?
i. के बबरामीहरुले दिर्घ रोगको बारेमा तपाईले भनेका कुरा सुन्छन र भाउ दिन्छन?
d. तपाईको आफ्नो ननयशमत काम बाहेक यो कामको लागग पयाघप्त समय छ?
e. के तपाईलाई यसबारे पयाघप्त ज्ञान र ताशलम छ िस्तो लाग्छ? वणघन गनुघहोस्
f. तपाईको पररवारको तपाइको काम प्रनत कस्तो धारणा छ?
g. यो कामको के कुराले तपाईमा ववतृष्णा पैिा गराउछ?
h. यो कामको के कुराले तपाईलाई उत्प्रेररत गराउछ?
i. तपाई र अन्य म.सा.स्वा.स्व./सा.स्वा.का.नेले आफ्नो अन्य काम र र्ायिी कामलाई कसरी समन्वय गनुघ हुन्छ?

7. **तपाईको ववचारमा के-कस्ता कुराले राम्रो म.सा.स्वा.स्व./सा.स्वा.का.िे िििाउछ िस्तो लाग्छ?**
a. के कुराले कुनै म.सा.स्वा.स्व./सा.स्वा.का.ने.ले राम्रो काम गरररराखेकी नछन् भन्ने बुखझन्छ?

i. नराम्रो काम?

b. राम्रो गनघको लागग म.सा.स्वा.स्व./सा.स्वा.का.ने. ले के कामहरु गनुघ पछघ होला ?

c. दिर्घ रोग व्यवस्थापन कायघिममा म.सा.स्वा.स्व./सा.स्वा.का.ने.को मापन र मुल्यािंकन गनघ सबैभन्िा उत्तम तररका के हुन सकछ?

8. **समुर्दायमा दर्दर्ा रोग व्यवस्थापिको िारेमा के पररवताि र्देख्ि चाहिहुन्छ िसले गर्दा सिैभन्दा िढी प्रभाव पिा सक्छ?**

a. तपाईलाई आफ्नो काम अझ राम्रोसिंग गनघको लागग?

b. कुन पररवतघनले बबरामी र वहाँहरुको पररवारमा सबैभन्िा बढी फाइिा गछघ?

कायघिम-पचचात ननिेशिका

१. **तपाईको समुर्दायमा दर्दर्ा रोगलाई कसरी हेररन्छ ?**

. के माननसहरुले यसको बारेमा सामान्यतया वा महत्वपूणघ छ भनेर हेछघन्?

i. दिर्घ र तीव्र रोगको बारेमा माननसहरुले कसरी एउटै वा शभन्न तररकाले सोच्छन्?

ii. दिर्घ रोग्ल्को उपचारमा परम्परागत वा बैकजल्पक उपचार प्रणालीको के भूशमका रहेको छ? के दिर्घ रोगकोलागग के यो उपचार प्रणाली कम वा बढी सान्धववघक छ?

. के सबैिाले यो रोगलाई एकै रुपमा हेछघन? कसरी?

. दिर्घकाशलन रोगले तपाईको समुिायमा केदह प्रभाव पारेको छ?

. दिर्घकाशलन रोगले तपाईको समुिायमा कसरी प्रभाव पारेको छ?

ङ. के बबरामीहरुलाई नतनीहरुको पररवारको राम्रो सहयोग रहन्छ? समुिायका अन्य माननसहरुबाट? के पररवार/समुिायका सबै रोगीलाई बराबर प्रभाव पिघछ?

. पररवारका सिस्य र समुिायका माननसले हेने दृस्टीकोणले बबरामीलाई कस्तो प्रभाव/असर पिघछ?

. यसले तपाईको काम गने उत्प्रेरणालाई कसरी प्रभाव पािघछ?

२. **समुर्दायका मानिसहरुलाई आफ्िो दर्दर्ा रोगको सफल व्यवस्थापिका चुिौनतहरु के के छि्?**

c. ती मध्ये कुन चादहँ सबैभन्िा बढी प्रभाव पािघछ? कुन चादहँ सबैभन्िा सामान्य/सधैं सामना गररररहनु पने हो?

d. कसलाई सबैभन्िा बढी र कसलाई सबैभन्िा कम प्रभाव पािघछ? कसरी? ककन?

i. मदहला, पुरुष, बालबाशलका, बृद्ध, मानशसक रोगग?

e. आफ्नो उपचारको िममा उनीहरुलाई कस्ता चुनौनतहरु सामना गनुघपछघ?

i. ननयशमत औषधी सेवन गनघ

1. बबरामीको औषगध सेवन गने बनन कसरी सुधानघ सककन्छ?

2. के पररवारले बबरामीलाई सम्झाउने वा िबाब दिने गछघ?

ii. बानीहरु सुधार गने, िस्तै – नुन, गचल्लो, गुशलयो कम खाने, चुरोट कम वपउने

iii. फलोअपको लागग ननयशमत आउने

f. बबरामीहरुलाई ननम्नहरुसिंग अन्तरकिया गनघ के चुनौतीहरु छन्:

i. स्वास््यकममीह – िस्तै डाकटर, नसघ, हेल्थ अशसस्टेन्ट, मदहला सामुिानयक स्वास््य स्वयिंसेववका ?

ii. स्वास््य सिंस्था र यसको व्यवस्थापनसिंग?

३. दर्दर्ा रोग फलोअप र रे-भ्रमणको लागग जिम्मेवार म.सा.स्वा.स्व./सा.स्वा.का.िेको भूशमकामा तपाई के चुिौती देख्िु हुन्छ?

g. तपाईको काममा के कुरा सबैभन्िा सजिलो छ?

h. तपाईको काममा के कुरा सबैभन्िा गाह्रो छ?

i. बबरामी र वहाँहरुको पररवारबाट के चुनौतीहरु सामना गनुघपछघ?

i. के बबरामीहरुले दिर्घ रोगको बारेमा तपाईले भनेका कुरा सुन्छन र भाउ दिन्छन?

j. तपाईको आफ्नो ननयशमत काम बाहेक यो कामको लागग पयाघप्त समय छ?

k. के तपाईलाई यसबारे पयाघप्त ज्ञान र ताशलम छ िस्तो लाग्छ? वणघन गनुघहोस्

l. तपाई र अन्य म.सा.स्वा.स्व./सा.स्वा.का.नेले आफ्नो अन्य काम र रीयिी कामलाई कसरी समन्वय गनुघ हुन्छ?

m. तपाईको पररवारको तपाइको काम प्रनत कस्तो धारणा छ?

n. यो कामको के कुराले तपाईमा ववतृष्णा पैिा गराउछ?

o. यो कामको के कुराले तपाईलाई उत्प्रेररत गराउछ?

p. के तपाई आफ्नो कामबाट सन्तुष्ट हुनुहुन्छ?

४. दर्दर्ा रोगका बिरामीहरुलाई म.सा.स्वा.स्व./सा.स्वा.का.िे.ले गरेको कामले कसरी प्रभाव पाछा िस्तो लाग्छ?

c. दिर्घ रोगका बबरामीको सहयोगको लागग म.सा.स्वा.स्व./सा.स्वा.का.ने ले खेल्ने भूशमकाको बारेमा के कुरा बबिेष छ िस्तो लाग्छ?

d. म.सा.स्वा.स्व./सा.स्वा.का.ने.को कुन कामले सबैभन्िा बढी प्रभाव पाछघ िस्तो लाग्छ?

e. सबैभन्िा कम प्रभाव?

f. के न्याय हेल्थका अन्य कमघचारीको काम सिंग खप्टीएको छ िस्तो लाग्छ?

g. ननम्न कुराहरुको बारेमा के धारणा छ:

i. बबरामीको स्व-व्यवस्थापन

ii. औषधी ननयशमत सेवन

iii. ननयशमत फलोअप

iv. रोग सम्बजन्ध बबरामीको बुझाई

५. अदहले न्याय हेल्थले गरररहेको दर्दर्ा रोग व्यवस्थापिको तररकाको िारेमा तपाईको के धारणा छ?

h. के यसलाई पूणघ सहयोग र तयारी छ?

i. के दिर्घ रोग व्यवस्थापन महत्वपूणघ छ? ककन छ वा छैन?

j. बबरामीको उपचारको सिंरचनाले गिाघ के बबरामीहरुलाई अप्ठ्यारो पारेको छ?

k. के उपचारको सिंरचनाले गिाघ तपाईहरुको काममा पनन चुनौनतहरु शसिघना भएका छन्?

l. म.सा.स्वा.स्व. र अन्य कमघचारी बबचको समन्वय र सहकायघको बारेमा के ववचार छ? म.सा.स्वा.स्व./सा.स्वा.का.ने, हेल्थ पोस्ट र अस्पतालको बबचको?

६. तपाईको ववचारमा के-कस्ता कुराले राम्रो म.सा.स्वा.स्व./सा.स्वा.का.िे िििाउछ िस्तो लाग्छ?

a. के कुराले कुनै म.सा.स्वा.स्व./सा.स्वा.का.ने.ले राम्रो काम गरररराखेकी नछन् भन्ने बुखझन्छ?

i. नराम्रो काम?

b. राम्रो गनघको लागग म.सा.स्वा.स्व./सा.स्वा.का.ने. ले के कामहरु गनुघ पछघ होला ?

c. दिर्घ रोग व्यवस्थापन कायघिममा म.सा.स्वा.स्व./सा.स्वा.का.ने.को मापन र मुल्याििंकन गनघ सबैभन्िा उत्तम तररका के हुन सकछ?

७. समुर्दायमा दर्दर्ा रोग व्यवस्थापिको िारेमा के पररवताि र्देख्ि चाहिहुन्छ िसले गर्दाा सिैभन्दा िढी प्रभाव पिा सक्छ?

d. तपाईलाई आफ्नो काम अझ राम्रोसिंग गनघको लागग?

e. कुन पररवतघनले बबरामी र वहाँहरुको पररवारमा सबैभन्िा बढी फाइिा गछघ?

कमघचारी अन्तरवाताघ ननिेशिका

कायघिम-पुवघको अन्तरवाताघ ननिेशिका

अन्तवाातााकारको लागग

सहभागीलाई न्याय हेल्थको हालको दिर्घ रोग व्यवस्थापन कायघिमको बारेमा बताउन लगाउनुस् र पनछ तलका प्रचनहरु सोध्न थाल्नु होला:

6. **न्याय हेल््मा तपाईले र्दर्दर्ा रोग स्याहारको के स्थाि र्देख्िु हुन्छ?**

a. के उनीहरुको स्याहारको सुधारको लागग सिंस्थागतरूपमा समथघन र उत्प्रेरणा प्राप्त छ िस्तो लाग्छ?

b. यस कायघिमलाई न्याय हेल्थ शभत्रका ववशभन्न समूह र ववभागहरुले कसरी शभन्न तररकाले हेछघन? स्वास््यकममीह र सहुिनयक स्वास््य ववभागले?

c. यो स्याहारले कस्तो प्राथशमकता राख्छ? तपाईको लागग, तपाईको ववभागको लागग, र अन्य ववभागको लागग?

7. **हालको प्रणालीले कजततको काम गरररराखेको छ िस्तो लाग्छ?**

a. यो कायघिम कनतको राम्रोसिंग सिंचाशलत छ? अनन व्यवजस्थत?

b. बबरामीको स्वास््य पररणामको मामलामा?

c. दिर्घकाशलन बबरामी व्यवस्थापनको मामलामा

i. औषगध

ii. फलोअप

iii. स्व-हेरचाह/व्यवस्थापन

iv. रोगको / लक्षणको ज्ञान

v. परामिघ

d. चुनौती, सबल पक्ष, पररवतघनका क्षेत्रहरु?

अन्तवाातााकारको लागग

दिर्घ रोग व्यवस्थापनको लागग योिना गररएका पररवतघनको बारेमा बताउनुहोस् |

 जकलननकल फलोअप र परामिघको बारेमा अझै बढी मानक *(standard)* प्रोटोकलहरु लागु गने

 सामुिानयक स्वास््य ववभागबाट परामिघलाई वविष िोड दिने –स्वास््यकममीहले दिर्घ रोगका बबरामीहरुलाई सा.स्वा.बब. पठाउनु पने

 हेल्थ पोस्टका कमघचारीलाई सानानतना दिर्घ रोगलाई अनुगमन, खोि, फलोअप, तथा प्रेषण गनघको लागग ताशलमको व्यवस्था गने

 दिर्घ रोगका बबरामीलाई फलोअपको लागग आउन सम्झाउन, स्वास््य परामिघलाई सुद्रीढ बनाउन, औषधी सेबनको बारेमा िान्न, तथा नयाँ बबरामीहरुको पदहचान गनघ म.सा.स्वा.स्व.लाई बबरामीको र्-र्मा पररचालन गने

 बबरामीको आफ्नो हेरचाह र रोग व्यवस्थापन आफैं गनघ िोड दिने

8. **सामुर्दानयक तहमा दर्दर्ा रोग व्यवस्थापि प्रणालीको सफल कायान्वयिको लागग के चुिौती र सिल पक्षहरु र्देखि्िुहुन्छ?**

a. एक बबरामीको लागग? ववशभन्न पररवार र समुिायका सिस्यको लागग कसरी शभन्न हुन्छ?

i. के बबरामीहरुले नतव्र र दिर्घ रोगको शभन्नता बुझ्छन?

१. यदि उनीहरुले आफ्नो लक्षण महसुस गनघ सक्िैनन् भने, यसले कसरी उनीहरुको सोचाईमा फरक पाछघ?

ii. ववशभन्न पररवारका सिस्यहरुको लागग:

१. मदहला, पुरुष, बालबाशलका, बृद्ध, मानशसक रोगग?

iii. ननम्न मामलाहरुमा:

१. औषगध

२. फलोअप

३. स्व-हेरचाह/व्यवस्थापन, रोगको / लक्षणको ज्ञान

४. परामिघ

iv. दिर्घ रोग व्यवस्थापनको लागग परम्परागत र बैकजल्पक उपचार ववगधको के स्थान छ? के त्यस्ता औषधीहरु दिर्घ रोग वा नतव्र रोगका लागग बढी उपयुकत हुन्छन्? के बबरामीहरुले त्यस्ता औषधीको प्रयोग गछघन्? के उनीहरुले त्यस्ता औषधीले अस्पतालका औषधीको सट्टामा प्रयोग गछघन्?

. के बबरामीहरुलाई उनीहरुको पररवार र समुिायले समथघन गछघन्?

i. कस्तो खालको समथघन?

ii. के शभन्न बबरामीलाई शभन्न खालको समथघन प्राप्त हुन्छ?

१. मदहला, पुरुष, बालबाशलका, बृद्ध, मानशसक रोगग?

. म.सा.स्वा.स्व./सा.स्वा.का.ने.को लागग:

i. ननम्न मामलामा:

१. कामको बोझ,

२. कामको प्राथशमकीकरण

३. भुकतानी

४. बबरशमसिंगको अन्तरककिया

५. क्षमता

६. सीप

ii. म.सा.स्वा.स्व. न्याय हेल्थका कमघचारी नभएका ले कसरी अनुगमन गने?
. समुिायको धारणा वा दृजष्टकोणको सन्िभघमा?
ङ. सामाजिक, सिंरचनात्मक, वन्िोवस्तीय, वा वातावरणीय ववषयहरूमा?
. के कस्ता अरु समुिायि स्रोतसाधनको पररचालन गरेर यस स्याहारलाई समथघन गनघ सककन्छ?
. सुधारको क्षेत्र?

9. संस्थागत तहमा दर्दर्ा रोग व्यवस्थापि प्रणालीको सफल कायान्वयिको लागग के चुिौती र सिल पक्षहरु देख्िुहुन्छ?

a. व्यवस्थापनको मामलामा?
b. अनुगमन र सुपररवेक्षणको मामलामा?
c. समन्वय र स्तर कायम गनघमा?
d. अस्पताल र सामुिानयक स्वास््य ववभाग बीच समन्वयमा?
i. ककन सा.स्वा.बब. र गचककत्सकहरु बबच राम्रो सिंवाि छैन?
ii. के गचककत्सकहरु सधैं ववरामीहरुलाई सा.स्वा.बब.मा पठाउछन्?
e. हेल्थपोस्टमा ढजल्कएकोमा?
i. नतनीहरू न्याय हेल्थका कमघचारी होइनन्?
ii. नतनीहरूको लागग कामका लागग समान अिाहरू वा उत्प्रेरणा नहुन सकछ
iii. हेल्थपोस्टका स्टाफको क्षमता र सीप?
iv. हेल्थपोस्ट र कमघचारीहरुमा सामुिायको ववचवास?
f. के परामिघले साँच्चै बबरामीको व्यवहार पररवतघन गनघ असर गछघ?
g. सुधारका क्षेत्र?

10. समुर्दायमा दर्दर्ा रोग व्यवस्थापि र रोकथामको सुधारको िारेमा के पररवताि देख्ि चाहिहुन्छ िसले गर्दा सिैभन्दा िढी प्रभाव पिा सक्छ?

a. तपाईलाई आफ्नो काममा?
b. न्याय हेल्थलाई?
c. बबरामीहरुको लागग?
d. त्यस्ता पररवतघनहरु कजत्तको व्यावहाररक हुन्छन्?
e. कुन सुधारले प्राथशमकता पाउनुपछघ र ककन?

कायघिम-पचचातको अन्तरवाताघ ननिेशिका

6. न्याय हेल््मा तपाईले दर्दर्ा रोग स्याहारको के स्थाि देख्िु हुन्छ?

a. के उनीहरुको स्याहारको सुधारको लागग सिंस्थागतरूपमा समथघन र उत्प्रेरणा प्राप्त छ िस्तो लाग्छ?
b. यस कायघिमलाई न्याय हेल्थ शभत्रका ववशभन्न समूह र ववभागहरुले कसरी शभन्न तररकाले हेछघन? स्वास््यकममीह र सहुिानयक स्वास््य ववभागले?
c. यो स्याहारले कस्तो प्राथशमकता राख्छ? तपाईको लागग, तपाईको ववभागको लागग, र अन्य ववभागको लागग?
d. यो कामको के महत्व छ?

7. सामुर्दानयक तहमा दर्दर्ा रोग व्यवस्थापि प्रणालीको सफल कायान्वयिको लागग के चुिौती र सिल पक्षहरु देख्िुहुन्छ?

a. एक बबरामीको लागग? ववशभन्न पररवार र समुिायका सिस्यको लागग कसरी शभन्न हुन्छ?
i. के बबरामीहरुले नतब्र र दिर्घ रोगको शभन्नता बुझ्छन?
१. यदि उनीहरुले आफ्नो लक्षण महसुस गनघ सकैनन् भने, यसले कसरी उनीहरुको सोचाईमा फरक पाछघ?
ii. ववशभन्न पररवारका सिस्यहरुको लागग:
१. मदहला, पुरुष, बालबाशलका, बृद्ध, मानशसक रोगग?
iii. ननम्न मामलाहरुमा:
१. औषगध
२. फलोअप
३. स्व-हेरचाह/व्यवस्थापन, रोगको / लक्षणको ज्ञान
४. परामिघ
. के बबरामीहरुलाई उनीहरुको पररवार र समुिायले समथघन गछघन्?
i. कस्तो खालको समथघन?
ii. के शभन्न बबरामीलाई शभन्न खालको समथघन प्राप्त हुन्छ?
१. मदहला, पुरुष, बालबाशलका, बृद्ध, मानशसक रोगग?
. म.सा.स्वा.स्व./सा.स्वा.का.ने.को लागग:
i. ननम्न मामलामा:
१. कामको बोझ,
२. कामको प्राथशमकीकरण
३. भुकतानी
४. बबरशमसिंगको अन्तरकिया
५. क्षमता
६. सीप
ii. म.सा.स्वा.स्व. न्याय हेल्थका कमघचारी नभएका ले कसरी अनुगमन गने?
ञ. समुिायको धारणा वा दृजष्टकोणको सन्िभघमा?
. सामाजिक, सिंरचनात्मक, वन्िोवस्तीय, वा वातावरणीय ववषयहरूमा?
. के कस्ता अरु समुिायि स्रोतसाधनको पररचालन गरेर यस स्याहारलाई समथघन गनघ सककन्छ?
. सुधारको क्षेत्र?

8. **संस्थागत तहमा दर्दर्ा रोग व्यवस्थापि प्रणालीको सफल कायान्वयिको लागग के चुिौती र सिल पक्षहरु देखि्िुहुन्छ?**

a. व्यवस्थापनको मामलामा?
b. अनुगमन र सुपररवेक्षणको मामलामा?
c. समन्वय र स्तर कायम गनघमा?
d. अस्पताल र सामुिानयक स्वास््य ववभाग बीच समन्वयमा?
i. ककन सा.स्वा.बब. र गचककत्सकहरु बबच राम्रो सिंवाि छैन?
e. हेल्थपोस्ट र म.सा.स्वा.स्व.मा ढजल्कएकोमा?
f. सुधारका क्षेत्र?

9. हालको स्याहार प्रणालीका अन्य मुख्य समस्याहरु के के िेखिहुन्छ?

a. ती समस्याहरु कसरी समाधान गनघ सककन्छ?

b. के हालको प्रणाली दिर्घ रोग स्याहारको लागग उजत्तकै व्यवजस्थत छ?

c. बबरामीको स्वास््य पररणामको मामलामा?

d. दिर्घकाशलन बबरामी व्यवस्थापनको मामलामा

i. औषगध

ii. फलोअप

iii. स्व-हेरचाह/व्यवस्थापन

iv. रोगको / लक्षणको ज्ञान

v. परामिघ

e. चुनौती, सबल पक्ष, पररवतघनका क्षेत्रहरु?

10. **समुर्दायमा दर्दर्ा रोग व्यवस्थापि र रोकथामको सुधारको िारेमा के पररवताि र्देख्ि चाहिहुन्छ िसले गर्दाा सिैभन्र्दा िढी प्रभाव पिा सक्छ?**

a. तपाईलाई आफ्नो काममा?

b. न्याय हेल्थलाई?

c. बबरामीहरुको लागग?

d. त्यस्ता पररवतघनहरु कजत्तको व्यावहाररक हुन्छन्?

e. कुन सुधारले प्राथशमकता पाउनुपछघ र ककन?

3. **Chronic disease electronic medical record templates (screenshots from electronic medical record system)**

Diabetes mellitus (type 2)

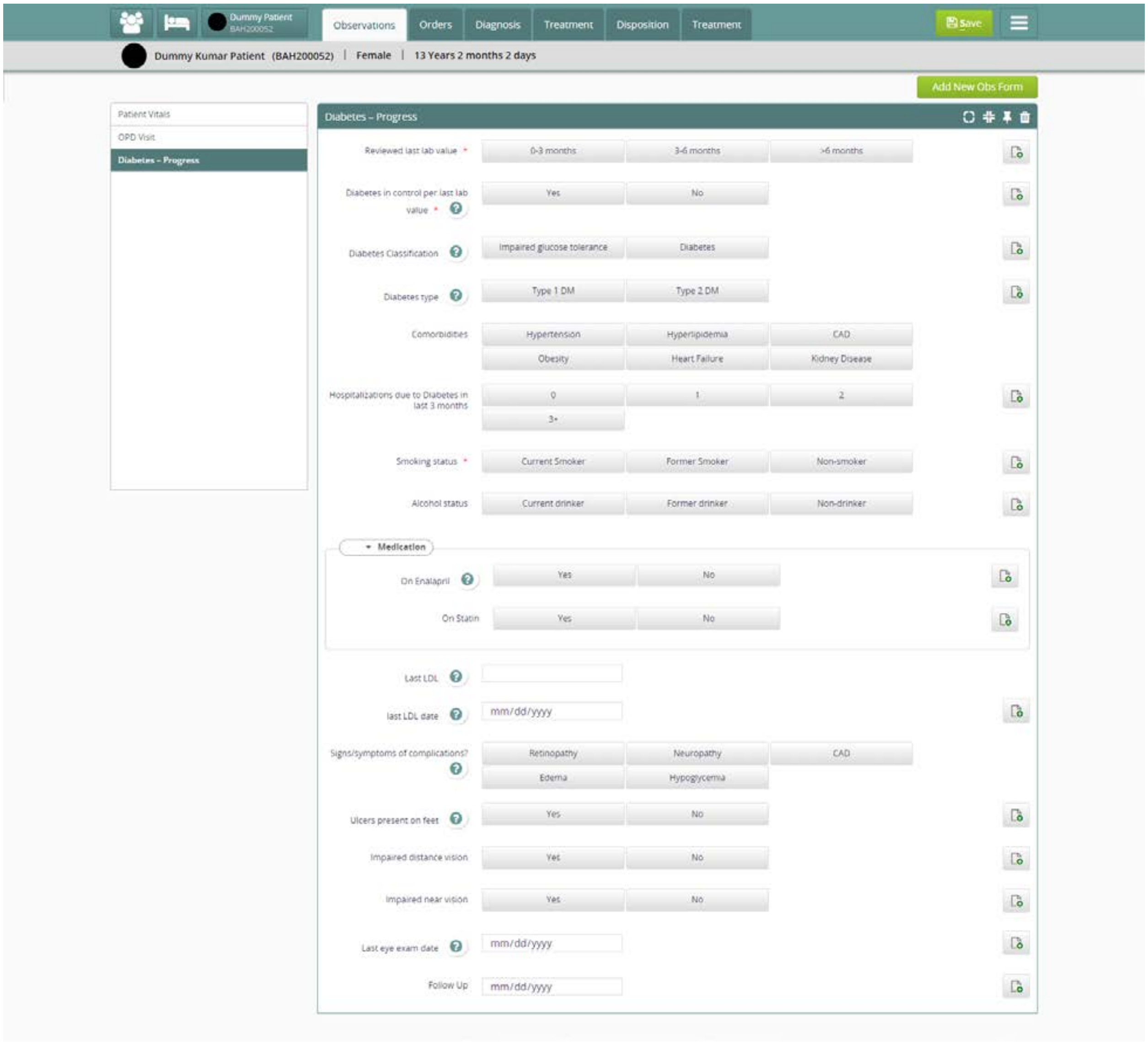

Dummy Patient
BAH200052
Observations
Orders
Diagnosis
Treatment
Disposition
Treatment
Save
Dummy Kumar Patient (BAH200052) | Female | 13 Years 2 months 2 days
Add New Obs Form
Patient Vitals
OPD Visit
Diabetes - Progress
Diabetes – Progress
Reviewed last lab value *
0-3 months
3-6 months
>6 months
Diabetes in control per last lab value *
Yes
No
Diabetes Classification
Impaired glucose tolerance
Diabetes
Diabetes type
Type 1 DM
Type 2 DM
Comorbidities
Hypertension
Hyperlipidemia
CAD
Obesity
Heart Failure
Kidney Disease
Hospitalizations due to Diabetes in last 3 months
0
1
2
3+
Smoking status *
Current Smoker
Former Smoker
Non-smoker
Alcohol status
Current drinker
Former drinker
Non-drinker
Medication
On Enalapril
Yes
No
On Statin
Yes
No
Last LDL
last LDL date
mm/dd/yyyy
Signs/symptoms of complications?
Retinopathy
Neuropathy
CAD
Edema
Hypoglycemia
Ulcers present on feet
Yes
No
Impaired distance vision
Yes
No
Impaired near vision
Yes
No
Last eye exam date
mm/dd/yyyy
Follow Up
mm/dd/yyyy

COPD

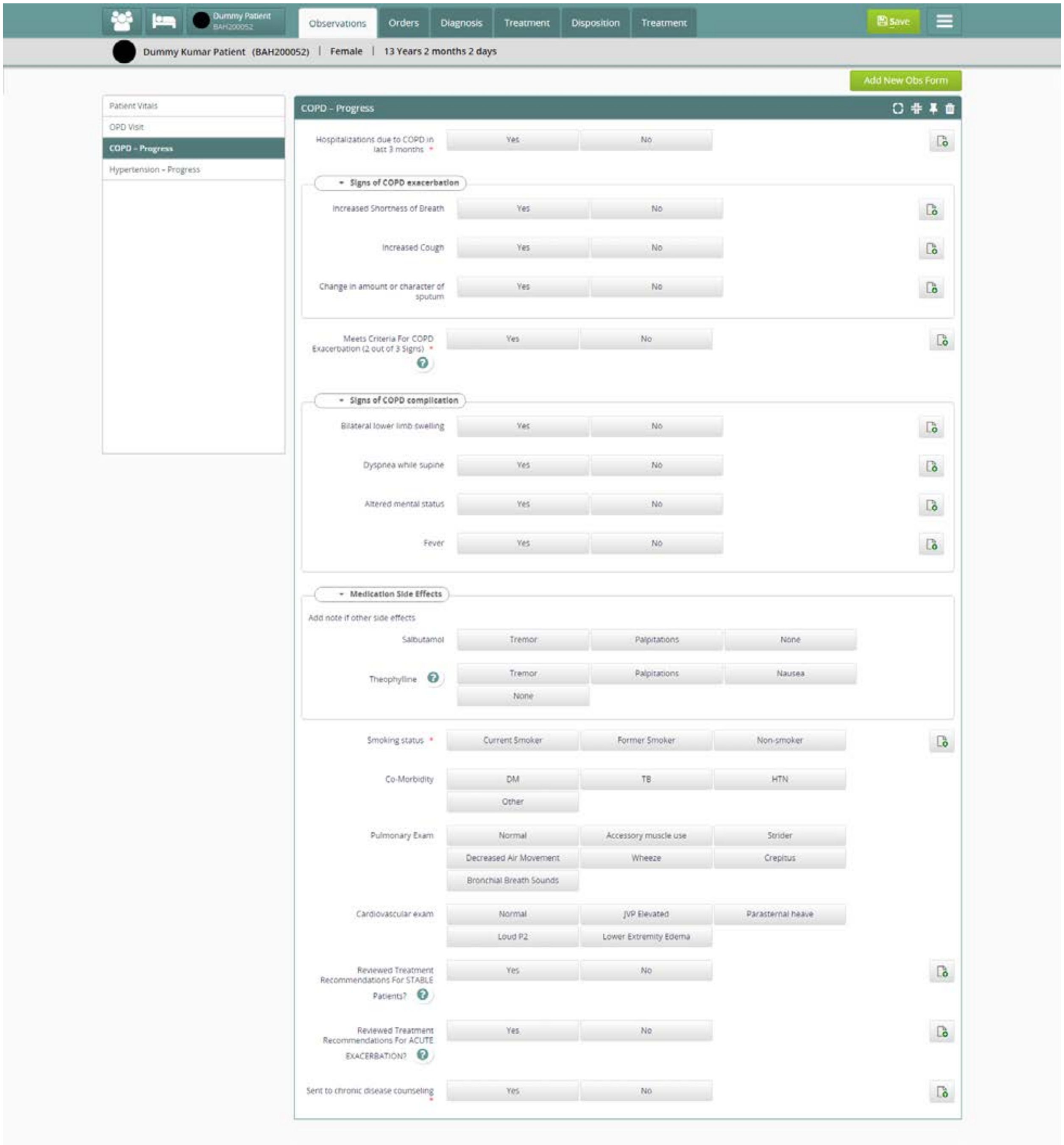

Dummy Patient
BAH200052
Observations
Orders
Diagnosis
Treatment
Disposition
Treatment
Save
Dummy Kumar Patient (BAH200052) | Female | 13 Years 2 months 2 days
Add New Obs Form
Patient Vitals
OPD Visit
COPD – Progress
Hypertension – Progress
COPD - Progress
Hospitalizations due to COPD in last 3 months *
Yes
No
Signs of COPD exacerbation
Increased Shortness of Breath
Increased Cough
Change in amount or character of sputum
Meets Criteria For COPD Exacerbation (2 out of 3 Signs) *
Signs of COPD complication
Bilateral lower limb swelling
Dyspnea while supine
Altered mental status
Fever
Medication Side Effects
Add note if other side effects
Salbutamol
Tremor
Palpitations
None
Theophylline
Nausea
Smoking status *
Current Smoker
Former Smoker
Non-smoker
Co-Morbidity
DM
TB
HTN
Other
Pulmonary Exam
Normal
Accessory muscle use
Strider
Decreased Air Movement
Wheeze
Crepitus
Bronchial Breath Sounds
Cardiovascular exam
JVP Elevated
Parasternal heave
Loud P2
Lower Extremity Edema
Reviewed Treatment Recommendations For STABLE Patients?
Reviewed Treatment Recommendations For ACUTE EXACERBATION?
Sent to chronic disease counseling *

Hypertension

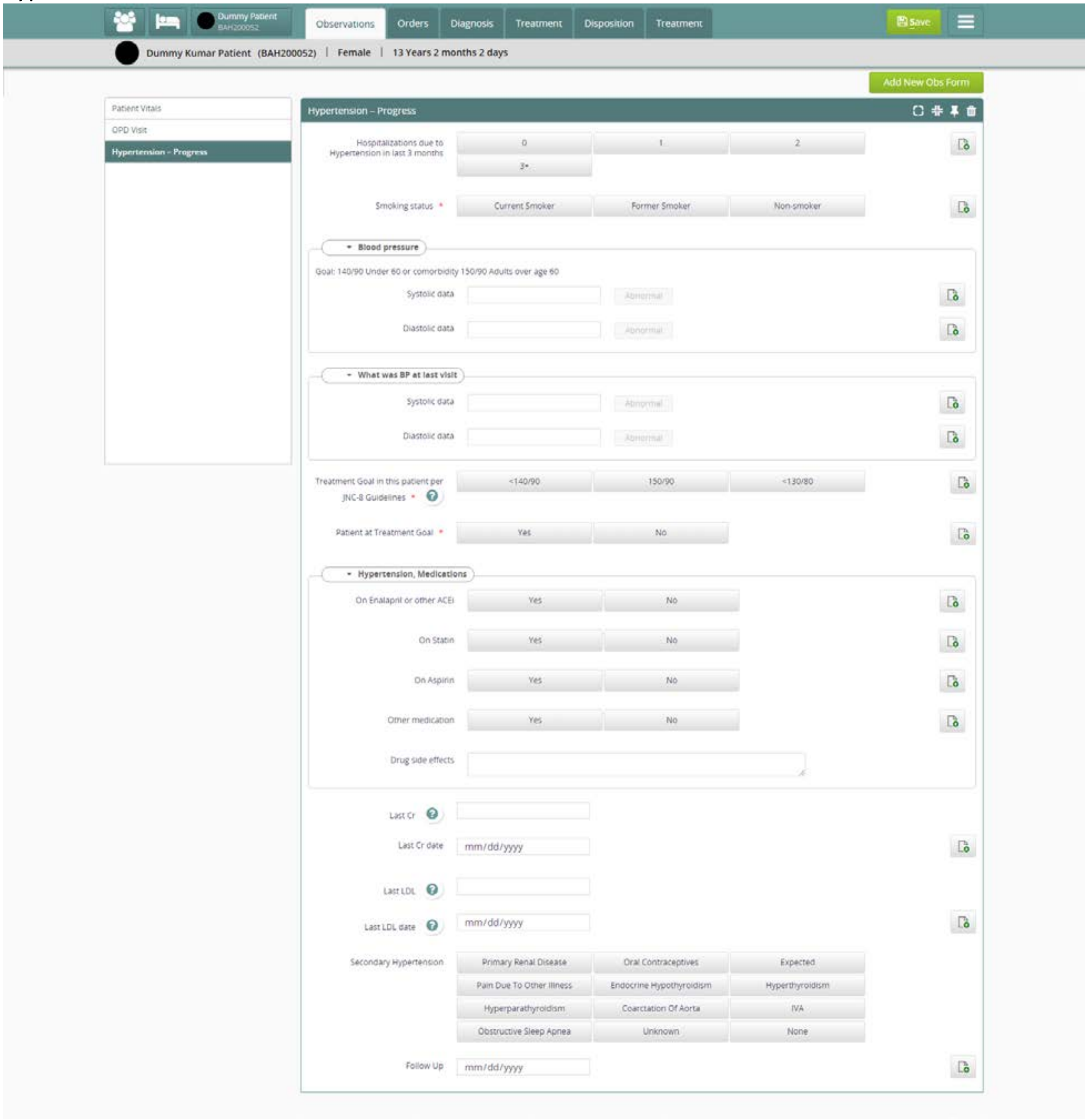

4. Risk factor patient interview guide

****(Main questions are numbered with suggested probes for exploring themes/topics following letters)*

1. Please can you start by describing your health at the moment? (COPD/Asthma: ask about respiratory health; Diabetes Mellitus/Hypertension: ask about physical health/nutrition).
    a. Do you feel your health has changed in recent years? Please can you describe any changes?

COPD/Asthma Pathway:

2. Now can you describe your current cooking practices?
    a. Can you think about your past cooking experience and describe how it was.
    b. How can you describe the differences between current and past cooking experience?

3. Now let's discuss on housing you are living in.
    a. Can you describe about the interior design of your housing?
    b. Please describe about the ventilation system in your kitchen and other rooms.
    c. Can you share your understanding on indoor air pollution?

4. Now let’s discuss about tobacco and smoking.
    a. Have your tobacco and smoking practices changed since learning you had COPD?
    b. Can you share your understanding of the relationship between tobacco/smoking and COPD?

5. Let us discuss on your understanding on Chronic Obstructive Pulmonary Diseases (COPD) the disease you are living with.
    a. In your opinion, how hard is it to be living with COPD in past years?
    b. Do you have knowledge on relation of [cooking practices and housing styles; tobacco and smoking] in causing COPD? Please describe.
    c. Can you share your other personal habits in daily basis?
    d. Other than medications, can you share some of the changes that you have perceived in your lifestyle to improve your health?

6. Can you share your ideas on remedies or preventive measures of COPD?
    a. What knowledge did you have about COPD before you got the disease?
    b. How have you been informed about COPD and other respiratory diseases in past? Was it through hospital or community health workers or any other medium?
    c. Have you been counseled [hospital/CHW] around risk factor modification [indoor cooking; tobacco and smoking]? How has this counseling been?
    d. In your opinion, how can this disease be prevented at personal and community level?
    e. What do you think about the effects of indoor air pollution on environment and how can it be controlled?

Diabetes Mellitus Pathway:

2. Now can you describe your current diet and nutrition practices?
    a. Can you think about your past dietary practices experience and describe how it was.
    b. How can you describe the differences between current and past diet?

3. Now let's us discuss on exercise.
    a. How physically active are you during the day.
    b. Can you describe about your exercise routine?
    c. How can you describe the differences between current and past exercise routines.

4. Let us discuss on your understanding on Diabetes Mellitus, the disease you are living with.
    a. In your opinion, how hard is it to be living with Diabetes in past years?
    b. Do you have knowledge on relation of diet and exercise in causing Diabetes? Please describe.
    c. Can you share your other personal habits in daily basis?
    d. Other than medications, can you share some of the changes that you have perceived in your lifestyle to improve your health?

5. Can you share your ideas on remedies or preventive measures of Diabetes?
    a. What knowledge did you have about Diabetes before you got the disease?
    b. How have you been informed about Diabetes in past? Was it through hospital or community health workers or any other medium?
    c. Have you been counseled [hospital/CHW] around risk factor modification [diet and nutrition; exercise]? How has this counseling been?
    d. In your opinion, how can this disease be prevented at personal and community level?

Hypertension Pathway:

2. Now can you describe your current diet and nutrition practices?
    a. Can you think about your past dietary practices experience and describe how it was.
    b. How can you describe the differences between current and past diet?
        c. [Sub-probe salt intake] Can you think about your past salt intake and describe how it was.
        d. [Sub-probe salt intake] How can you describe the difference between current and past salt intake?

3.. Now let's discuss about tobacco and smoking.
    a. Have your tobacco and smoking practices changed since learning you had hypertension?
    b. Can you share your understanding of the relationship between tobacco/smoking and hypertension?

4. Let us discuss on your understanding on Hypertension, the disease you are living with.
    a. In your opinion, how hard is it to be living with Hypertension in past years?
    b. Do you have knowledge on relation of diet and exercise in causing Hypertension? Please describe.
    c. Can you share your other personal habits in daily basis?
    d. Other than medications, can you share some of the changes that you have perceived in your lifestyle to improve your health?

5. Can you share your ideas on remedies or preventive measures of Hypertension?
    a. What knowledge did you have about Hypertension before you got the disease?
    b. How have you been informed about Hypertension in past? Was it through hospital or community health workers or any other medium?
    c. Have you been counseled [hospital/CHW] around risk factor modification [indoor cooking; tobacco and smoking]? How has this counseling been?
    d. In your opinion, how can this disease be prevented at personal and community level?
